# Responses of the Macroalga *Ulva prolifera* Müller to Ocean Acidification Revealed by Complementary NMR- and MS-Based Omics Approaches

**DOI:** 10.3390/md20120743

**Published:** 2022-11-26

**Authors:** Carlos Sanchez-Arcos, Debora Paris, Valerio Mazzella, Mirko Mutalipassi, Maria Costantini, Maria Cristina Buia, Eric von Elert, Adele Cutignano, Valerio Zupo

**Affiliations:** 1 Institute for Zoology, Cologne Biocenter University of Cologne, 50674 Köln, Germany; 2 Consiglio Nazionale delle Ricerche (CNR), Istituto di Chimica Biomolecolare (ICB), 80078 Pozzuoli, Italy; 3 Department of Integrative Marine Ecology, Stazione Zoologica Anton Dohrn, Ischia Marine Center, 80077 Ischia, Italy; 4 Department of Integrative Marine Ecology, Stazione Zoologica Anton Dohrn, Calabria Marine Centre, 87071 Amendolara, Italy; 5 Department of Ecosustainable Marine Biotechnology, Stazione Zoologica Anton Dohrn, 80121 Napoli, Italy; 6 Department of Ecosustainable Marine Biotechnology, Stazione Zoologica Anton Dohrn, 80077 Ischia, Italy

**Keywords:** ocean acidification (OA), *Ulva prolifera*, metabolomics, lipidomics, LC-MS, NMR, macroalgae

## Abstract

Ocean acidification (OA) is a dramatic perturbation of seawater environments due to increasing anthropogenic emissions of CO_2_. Several studies indicated that OA frequently induces marine biota stress and a reduction of biodiversity. Here, we adopted the macroalga *Ulva prolifera* as a model and applied a complementary multi-omics approach to investigate the metabolic profiles under normal and acidified conditions. Our results show that *U. prolifera* grows at higher rates in acidified environments. Consistently, we observed lower sucrose and phosphocreatine concentrations in response to a higher demand of energy for growth and a higher availability of essential amino acids, likely related to increased protein biosynthesis. In addition, pathways leading to signaling and deterrent compounds appeared perturbed. Finally, a remarkable shift was observed here for the first time in the fatty acid composition of triglycerides, with a decrease in the relative abundance of PUFAs towards an appreciable increase of palmitic acid, thus suggesting a remodeling in lipid biosynthesis. Overall, our studies revealed modulation of several biosynthetic pathways under OA conditions in which, besides the possible effects on the marine ecosystem, the metabolic changes of the alga should be taken into account considering its potential nutraceutical applications.

## 1. Introduction

Atmospheric concentrations of carbon dioxide are progressively increasing and the consequent reduction of pH in water basins prompts direct and indirect effects on the metabolism of marine organisms, leading to demonstrated changes in coastal ecosystems, sometimes overlapping with additional stressors such as pollution, eutrophication, and riverine discharges [1,2,3]. The process appeared continuous and consistent in the last decades and empirical models indicate that carbon dioxide concentrations will increase in the atmosphere from the present levels (about 412.15 ppm) up to about 750 ppm by the end of this century [4]. Consequently, the pH of oceans could decrease to 7.8–7.7 in parallel, leading to well-known effects on benthic communities at various levels [5]. In fact, the absorption of CO_2_ has already caused apparent changes in the carbonate chemistry of surface seawater and is predicted to decrease by 0.44 ± 0.005 units from 1870–1899 to 2080–2099, under SSP5-8.5 scenarios (IPCC, 2021) [4]. Overall, ocean acidification (OA) affects the biology of the oceans through direct effects on calcifying organisms and indirect effects on benthic and planktonic communities [6]. Direct effects such as the lack of development of coral reefs under pH 7.7 and the impairment of physiology for calcifying animals and coralline algae have been clearly demonstrated [6,7,8,9]. However, various authors still debate the possible consequences, according to various levels of pH representing thresholds for the partial or total destruction of coral reefs [10,11].

Nevertheless, positive effects are forecasted for selected organisms because a higher abundance of carbon dioxide may induce faster growth of algae, changes in the chemical composition of seagrasses, and consequently, larger resources available for some herbivores [12,13,14]. Although CO_2_ is an essential source of nutrients for photosynthesis, the benefits for autotrophs largely depend on the photosynthetic pathways evolved. For example, C4 plants adopt phosphoenolpyruvate carboxylase, and they experience minor photorespiratory losses due to the use of carbon-concentrating mechanisms [15]. Consequently, they might not benefit from increased levels of CO_2_ because phosphoenolpyruvate carboxylase is substrate-saturated at the levels of CO_2_ currently experienced in seawater.

In contrast, C3 plants that use RuBisCO as the priming carboxylation enzyme might experience a loss of fixed carbon due to photorespiration and benefit from seawater acidification. RuBisCO is not substrate-saturated at the levels of carbon presently measured in seawater [15]. These consequences lead to complex scenarios of ecosystem modifications, including changes in biodiversity and community composition [8,10,16,17]. For example, Garrard et al. [17] indicated clear differences in the community structure of seagrasses, driven by the indirect effects of acidification, such as changes to canopy structure and food availability, rather than physiological intolerance to low pH levels. The number of invertebrates collected in acidified stations was almost double that of control stations during the study and many heavily calcified species appeared to thrive. All this led to changes in community structure and biodiversity. Other investigations demonstrated that the levels of biodiversity in marine benthic communities are strongly impacted by ocean acidification and temperature rises [18]. In addition, it was also observed that the loss of habitat complexity of coral reefs in response to natural acidification led to a decrease in some groups of mobile animal taxa and that the overall biodiversity was reduced [19]. Previous research tried to forecast future consequences in various environments, considering the levels of ecosystem vulnerability [20]. Heat stress and other impacts (e.g., industrial pollution, effects of fisheries, touristic activities, coastal urbanization, increases of turbidity, discharges of drugs from aquaculture plants) complicate the process and lead to variable predictions about key environments subjected to multi-stressor conditions [7]. In addition, indirect effects of the OA, such as the impacts on infochemical communications among organisms, may encompass the direct effect of the pH on the phenotype of plants and animals because the adaptation of organisms to a given environment is a complex process involving chemical defenses and communications that can be impaired even by minor changes of pH [21,22,23]. However, only few metabolomic studies are dedicated to macroalgae, despite their ecological importance in marine ecosystems [24,25,26,27,28].

As mentioned, calcifying algae are severely threatened by OA due to its direct effects on their external tissues [23,29]. However, non-calcified algae could also be impacted because changes in their composition might be expected [30]. Among Ulvaceae, the alga *Ulva rigida* was investigated under increased pCO_2_ and a clear preference and substantial HCO_3_^−^ use was suggested to support photosynthesis and growth [31]. Therefore, increased levels of CO_2_ predicted for the future are not likely to stimulate *Ulva rigida* blooms [32]. Considering that OA alters the carbon speciation in seawater, this influence has implications for photosynthesis and C:N ratios of primary producers [33]. A wide range of algal responses to OA has been observed, including both positive effects on their growth and negative impacts on their ecology, which may be explained by differences in algal physiology, timescales of the responses measured, and specific metabolic features of individual species. However, some generic responses may be expected and modeled using another widely diffused alga, such as *Ulva prolifera*. This species is almost ubiquitous and has been suggested to be an excellent model for predicting seaweed growth in light- and nutrient-limited environments and its tissue modifications due to environmental changes [34]. *U. prolifera* (previously known as *Enteromorpha prolifera*) is a marine macroalga with medicinal and edible potential, but congeneric algae are also the cause of undesirable green tides which seriously impact the coastal ecology and the environment in various regions. The effects of the temperature on the metabolic pathways of this alga have been investigated and it was demonstrated that the metabolic profiles of *U. prolifera* cultured under high-and low-temperature treatments were significantly different [35]. This alga is often responsible for “green tides” in various areas of the world and may exhibit morphological variations according to environmental conditions and developmental stages [36]. On the other hand, *U. prolifera* is a macroalga characterized by high palatability and high levels of microelements, making it an important food item for marine herbivores and a candidate additive for animal feeds [37]. It has been widely used as an algal model to detect physiological changes due to various stresses by means of metabolomic investigations [38,39].

Environmental metabolomics and lipidomics have emerged as powerful approaches to studying organisms’ chemical responses and adaptations to specific environmental conditions [40,41,42]. In marine environments, the metabolism of aquatic organisms is continuously affected by exposure to abiotic stressors, such as changes in temperature [43,44], light intensity [45], oxygen levels [46], and salinity [47]. A summary of their functional metabolic phenotype would contribute to understanding the chemical mechanisms underpinning the adaptations to these stressors [48]. Liquid chromatography–mass spectrometry (LC-MS)- and nuclear magnetic resonance (NMR)-based metabolomics/lipidomics have steadily evolved during the last decade, allowing the elucidation of a greater variety of metabolites in complex biological samples and providing sufficient information to directly determine active biochemical pathways. Although some studies have already assessed the metabolic changes of marine organisms due to OA, information about the chemical adaptations of marine algae is still incomplete [24,25,26,27,28,48,49,50,51].

Here, we applied a multidisciplinary approach to investigate the growth rates and the metabolic responses of the common and ubiquitous marine macroalga, *U. prolifera* Müller, under normal and acidified conditions, hypothesizing that increasing levels of CO_2_ could modify the growth and the chemical turnover of this alga. We aim to elucidate the multivariate effects of OA on this marine macroalga’s growth and metabolic profiles. In particular, we monitored the growth and performed a complementary NMR- and LC-MS-based metabolomics and lipidomics analysis after 55 days of cultivation of *U. prolifera* in special photobioreactors, facilitating the tuning of the pH conditions to detect changes in its chemical profiles that could affect its resilience and nutritional quality for grazers. Comparing metabolomics and lipidomics data permits obtaining complementary insights into the effects of OA on macroalgae and allows us to speculate about how these effects may influence the shaping of benthic communities and their physiologic and trophic relationships.

## 2. Results

### 2.1. Macroalgal Growth in Normal and Acidified Conditions

**Figure 1:**
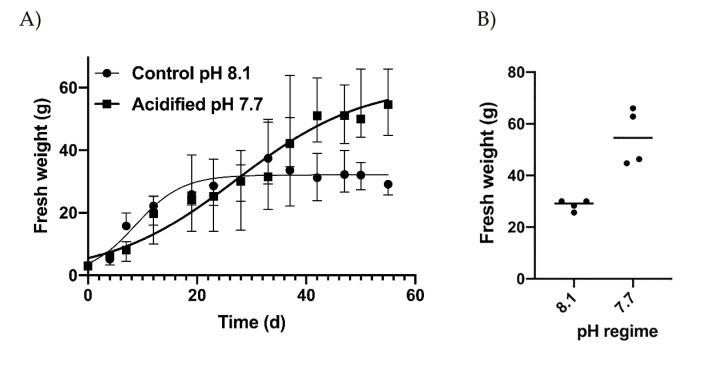
(**A**) Growth patterns in control (pH 8.1) and acidified (pH 7.7) conditions expressed as mean values ± SD, and (**B**) final fresh weight of algae obtained at the two pH regimes. Dots indicate the replicates. The horizontal lines indicate the mean values.

Under normal conditions (pH 8.1), exponential growth was observed immediately after the start of the experiment. A stationary phase was reached after about 20 days of culture, keeping the average weight of thalli at 31.56 g (±2.76), while in acidified conditions, a lower slope but a continuous exponential growth up to the end of the experiment (Figure 1A), when thalli reached an individual fresh weight of 59.98 g (±10.97), occurred. The final weights obtained in acidified conditions were significantly higher (*t*-test, *p* < 0.05). The parameters of the growth equations were quite different (Supplementary Table S1) as well, with *Y*_m_ and *X*_int_ in acidified conditions reaching two times higher levels. However, the Mann-Whitney test indicated non-significant differences between the two curves due to high variation among replicates. In particular, large variation characterized the replicates obtained in acidified conditions, while the final weights obtained in normal conditions were almost consistent in all replicates (Figure 1B). The slopes of the two growth curves obtained for normal and acidified conditions were significantly different (Figure 1A) at *p* < 0.01 (confidence intervals evaluated by GraphPad Prism, version 8, San Diego, CA, USA).

### 2.2. NMR-Based Metabolomics

#### 2.2.1. NMR Analysis on Polar Compounds

**Figure 2:**
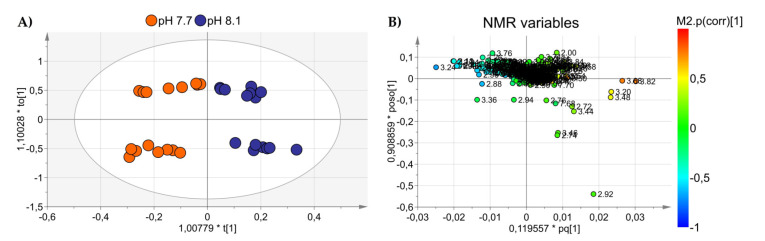
(**A**) OPLS-DA score plot showing NMR spectra projections of alga samples at pH 7.7 (orange dots) and pH 8.1 (blue dots) discriminated along with the first component. (**B**) Loadings are associated with the score plot in (**A**) and indicate the variables responsible for data discrimination.

**Figure 3:**
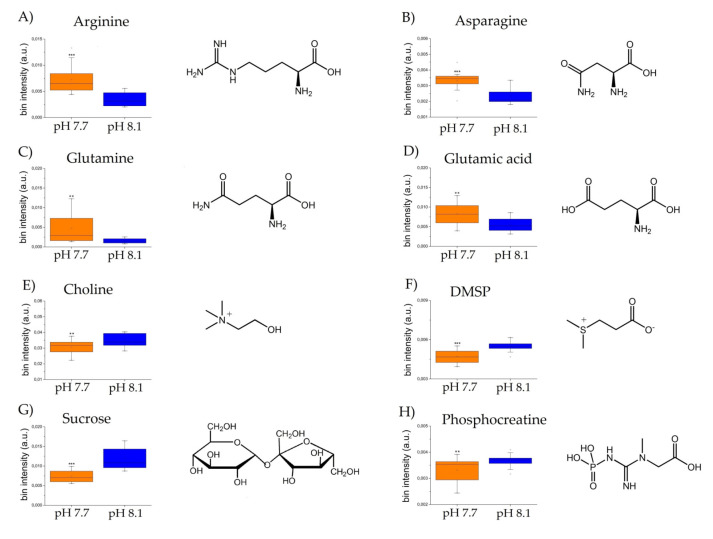
Box and Whisker’s plot of normalized bins showing the levels of discriminant metabolites of alga samples at pH 7.7 (orange) and pH 8.1 (blue) obtained by NMR analyses: (**A**–**D**) up-modulated metabolites, and (**E**–**H**): down-modulated metabolites. Student’s *t*-test significance is reported as: *p*-values = *** <0.001, ** <0.01.

An OPLS-DA was applied to visualize the changes in the algal polar profiles in response to acidification. An excellent statistical model was obtained with one predictive and two orthogonal components and parameters *R*^2^ = 0.81, *Q*^2^ = 0.63, and CV-ANOVA *p* value = 0.0001, representing the goodness-of-fit, the goodness-of-prediction, and the estimation of the ANOVA testing of cross-validated predictive residuals, respectively. As shown in the score plot of Figure 2A, samples from different pH levels are discriminated along the predictive component *t*(1), while the orthogonal component mainly accounts for intra-class inhomogeneity. In particular, the pH 8.1 group is placed at *t*(1) positive coordinates, while the acidified class is projected at *t*(1) negative values. By inspecting the associated loading plot of Figure 2B and assigning the corresponding molecules, we found that high levels of the amino acids characterized the group at pH 7.7: glutamine (2.46 ppm, 2.44 ppm), glutamate (2.34 ppm, 2.16 ppm), arginine (3.24 ppm, 1.90 ppm, 1.74 ppm, 6.90–6.92 ppm), and asparagine (2.96 ppm, 2.88 ppm), while the control group at pH 8.1 exhibited higher concentrations of choline (3.20 ppm), DMSP (3.48–3.50 ppm), sucrose (5.40–5.42 ppm, 3.82 ppm), and phosphocreatine (3.04 ppm) (Figure 3). Overall, a total of 367 features were evaluated, and 40 were found dysregulated (|pcor| > 0.5), corresponding to a total of 12 polar metabolites, 8 of which were found statistically significant in discriminating the two algal experimental sets (*p* ≤ 0.05).

#### 2.2.2. Metabolic Pathway Analysis

**Figure 4:**
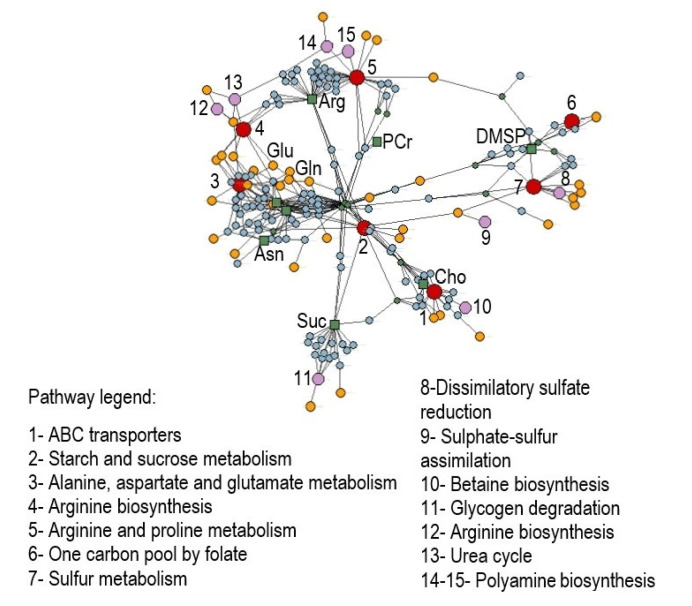
Enrichment analysis on discriminant polar metabolites obtained by NMR analyses that differentiate *Ulva prolifera* under acidification conditions. Networks including pathways (1–7, red dots), modules (8–15, pink dots), enzymes (yellow dots), reactions (cyan dots), and metabolites (green dots and squares) were elaborated using label propagation in a knowledge model network based on the *Chlamydomonas reinhardtii* database in KEGG (*p* < 0.05). Abbreviations: Cho = choline; Suc = succinate; Asn = asparagine; Gln = glutamine; Glu = glutamate; Arg = arginine; PCr = phosphocreatine; DMSP = dimethylsulfoniopropionate.

Metabolic pathway enrichment analysis of significantly modulated metabolites was performed according to the ‘diffusion’ algorithm based on annotated metabolites’ information in the KEGG database related to the green alga *Chlamydomonas reinhardtii*, where every metabolite can map the term, including the module, pathway, metabolites, enzyme, and reaction, among others. As a result, a total of 197 nodes were found by the analysis with a *p*-value set to 0.05, consisting of 7 related pathways, 8 modules, 39 enzymes, 122 reactions, and 13 additional metabolites found to be involved (ATP, ADP, orthophosphate, glucose, aspartate, fructose, tetrahydrofolate, creatine, 5-methyltetrahydrofolate, acrylic acid, dimethyl sulfide, creatinine, and 3-methylthiopropanoate), besides the input ones (arginine, asparagine, glutamine, glutamate, choline, phosphocreatine, sucrose, and DMSP) (Figure 4 and Table S2).

### 2.3. LC-MS-Based Metabolomics

**Figure 5:**
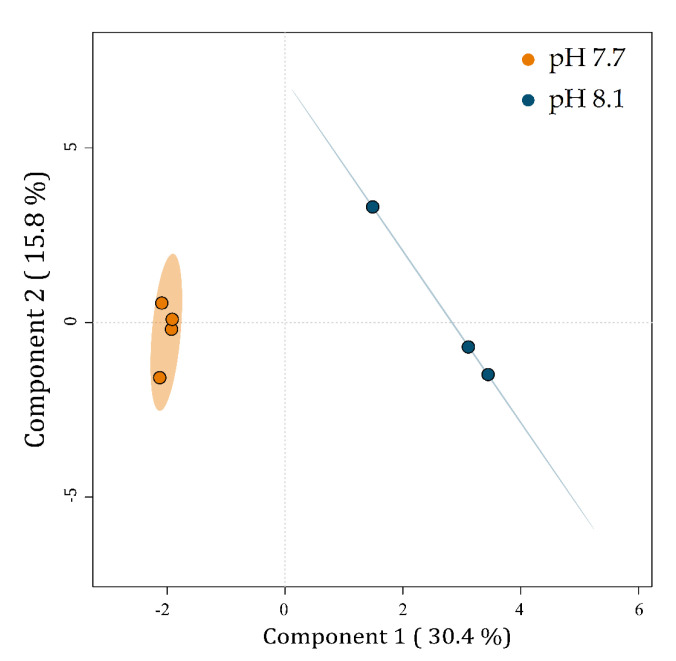
PLS-DA plot of the LC-MS-based metabolic profiles of *Ulva prolifera* cultivated at different pH levels. Small colored circles represent individual metabolic profiles of specimens cultivated at pH 7.7 (orange) and 8.1 (blue). Colored ellipses represent the 95% confidence regions for each group.

A PLS-DA was carried out to visualize the relationships between the metabolic profiles of *U. prolifera* cultivated at two pH levels. The PLS-DA plot reveals discrimination between the metabolic profiles of algae cultivated at pH 7.7 from those at 8.1 (Figure 5).

Component 1 explains 30.4% of the data variability and separates the metabolic profiles of *U. prolifera* cultivated at pH 7.7 on the left from those at pH 8.1 on the right. Component 2 explains 15.8% of the total variability of the data but does not separate the groups.

#### 2.3.1. Identification of Modulated Metabolites

**Figure 6:**
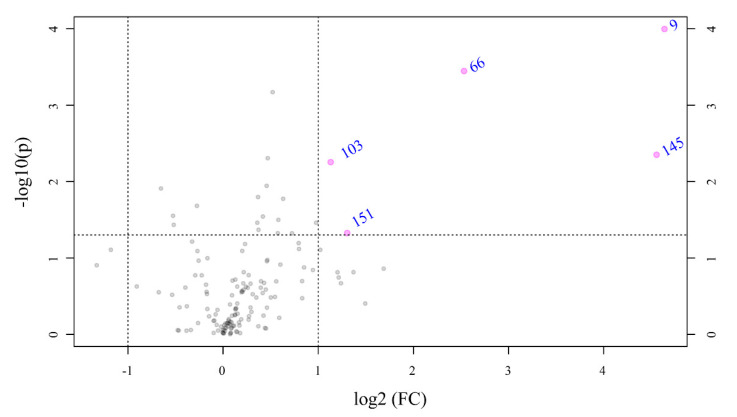
Volcano plot based on the LC-MS analyses showing metabolites with fold changes (FC) ≥ 2.0 (*p* ≤ 0.05) in *Ulva prolifera* when the pH cultivation media was modified from 8.1 to 7.7 (FC = amount at pH 7.7/amount at pH 8.1). The plot shows the −log 2 of the amount of each metabolite at pH 7.7 with respect to the amount of the same compound at pH 8.1. The pink circles and names in blue indicate metabolites with FC ≥ 2.0 at *p* ≤ 0.05. The data presented correspond to the statistical analysis of four biological replicates at pH 7.7 and three at pH 8.1.

**Figure 7:**
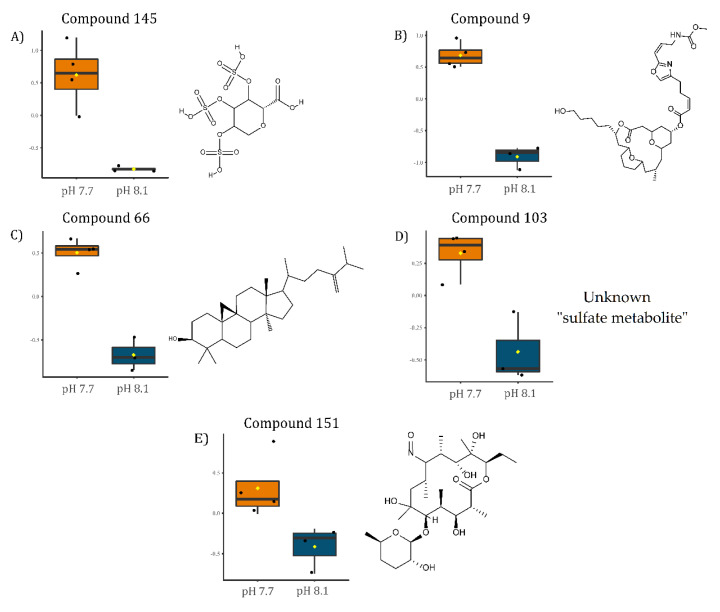
Boxplots of the relative levels of: (**A**) (2*R*)-3,4,5-Trisulfooxyoxane-2-carboxylic acid, (**B**) [(3*R*,7*R*,13*S*)-13-(5-hydroxypentyl)-3-methyl-11-oxo-12,19,20-trioxatricyclo[13.3.1.15,9] icosan-7-yl] (*Z*)-5-[2-[(*Z*)-3-(methoxycarbonylamino)prop-1-enyl]-1,3-oxazol-4-yl]pent-2-enoate, (**C**) 24-methylene-cycloartanol, (**D**) unknown sulfate metabolite, and (**E**) (*Z*)-3-[4-[[3-(dimethylamino)-5-methyloxan-2-yl]-hydroxymethoxy]propan-2-yl]-3,9-dihydroxy-6,8-dimethyl-10-oxodec-4-enoic acid. Chemical structures on the right side of each boxplot represent the putative identification for each compound.

To identify the significantly modulated metabolites, a volcano plot comparing pH 7.7/8.1 was executed (FC ≥ 2.0, *p*-value ≤ 0.05) (Figure 6). A total of 106 metabolites appeared modulated in the algal samples under acidified conditions (pH 7.7) vs. normal conditions (pH 8.1). Five metabolites were significantly upmodulated in *U. prolifera* (Figure 7). All these compounds were putatively identified by comparing their MS/MS spectra acquired with those available in tools that combine free compound database searching and fragmentation prediction. Compound **145** (Figure 7A and Supplementary Figure S1) was identified as (2R)-3,4,5-Trisulfooxyoxane-2-carboxylic acid. Compound **9** (Figure 7B and Supplementary Figure S2) was identified as an oxazole-containing macrolide. Compound **66** (Figure 7C and Supplementary Figure S3) was identified as 24-methylene-cycloartanol. Compound **103** (Figure 7D and Supplementary Figure S4) is a sulfate metabolite whose structure remains unknown. Compound **151** (Figure 7E and Supplementary Figure S5) was identified as the macrolide derivative (*Z*)-3-[4-[[3-(dimethylamino)-5-methyloxan-2-yl]-hydroxymethoxy]propan-2-yl]-3,9-dihydroxy-6,8-dimethyl-10-oxodec-4-enoic acid.

### 2.4. LC-MS-Based Lipidomics

**Figure 8:**
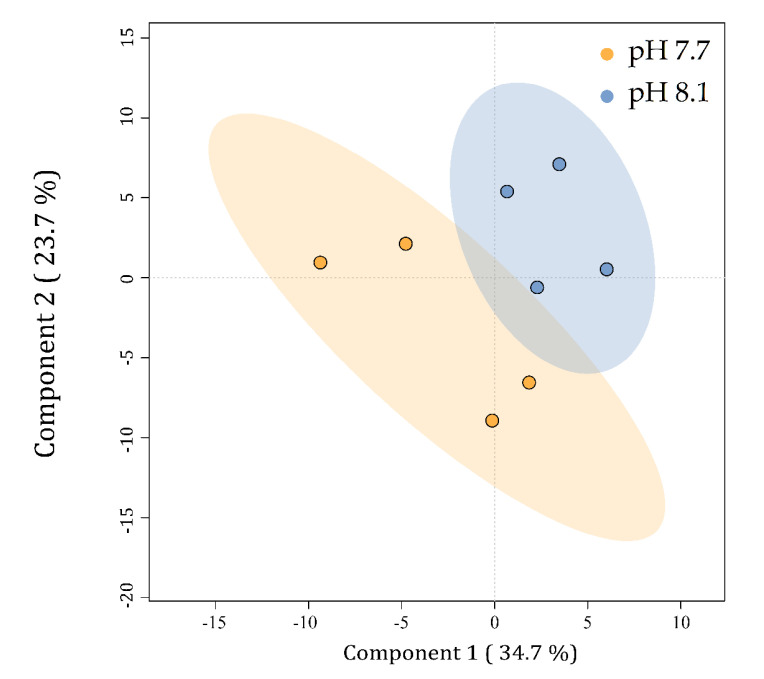
PLS-DA plot of the lipid profiles of *Ulva prolifera* cultivated at different pH levels. Small colored circles represent single algal lipid profiles at pH 7.7 (orange) and 8.1 (blue). Colored ellipses represent the 95% confidence regions for each group.

Lipid yield was measured in both conditions and showed a significant (*p* < 0.05) increase at pH 7.7 (5.04% ± 0.38% DW) as compared to a normal growth at pH 8.1 (4.23% ± 0.34% DW) (Supplementary Figure S6). Preliminary insight into the lipid class profile, based on ion intensities, indicated neutral lipids (TG) and glycolipids (sulfolipids SQDG and SQMG and galactolipids MGDG, MGMG, DGMG) as the most abundant species in *U. prolifera*, followed by phospholipids (PI, PC, PE, and PG) and ceramides (Supplementary Figure S7). Biological replicates were individually considered for statistical analyses, while technical replicates were averaged. To visualize the relationships between the lipid profiles of *U. prolifera* cultivated at different pH levels, a partial least square discriminant analysis (PLS-DA) was carried out. The PLS-DA plot revealed differences between the lipid profiles of cells cultivated at pH 7.7 from those at 8.1 (Figure 8). The replicates within each batch, particularly in acidified conditions, clustered into two quite divergent subgroups, reflecting the behavior shown for the algal growth curve (Figure 1). Component 1 explains 34.7% of the data variability and discriminates the metabolic profiles of the alga cultivated at pH 7.7 (on the left) from those at pH 8.1 (on the right). Component 2 explains 23.7% of the data’s total variability but does not discriminate between the groups. Although no statistically relevant variation in lipid class/species was detected, the discrimination observed in the PLS-DA indicated that for *U. prolifera,* the acidification of the growing conditions modulated the lipid profiles.

#### 2.4.1. Identification of Modulated Lipids

**Figure 9:**
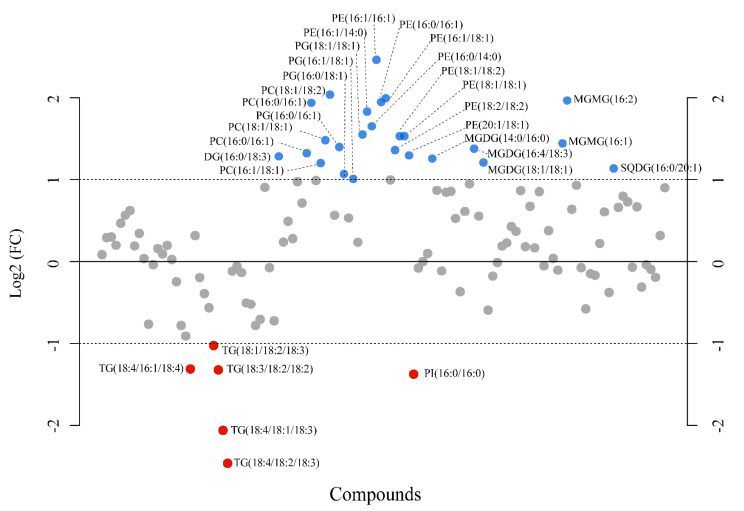
Fold-change analysis plot showing lipids with fold changes (FC) ≥ 2.0 in *Ulva prolifera* when the pH cultivation media was modified from 8.1 to 7.7 (FC = amount at pH 7.7/amount at pH 8.1). The plot shows the −log 2 of the amount of each metabolite at pH 7.7 with respect to the amount of the same species at pH 8.1 per mg of dry alga. Blue and red circles indicate lipids upmodulated and down-modulated, respectively. The data presented correspond to the analysis of four biological replicates for each experimental condition.

A fold-change analysis was executed comparing pH 7.7 and 8.1 (FC ≥ 2.0) and a total of 116 lipids were found modulated; however, only 31 metabolites fulfilled the criteria of being modulated with a FC ≥ 2, in particular, 25 upmodulated and 6 down-modulated lipids (Figure 9). Among the upmodulated lipids, the phospholipids PC, PG, and PE were the predominant classes (Supplementary Figures S8–S10). On the other hand, TG was the most down-modulated lipid class due to acidification.

**Figure 10:**
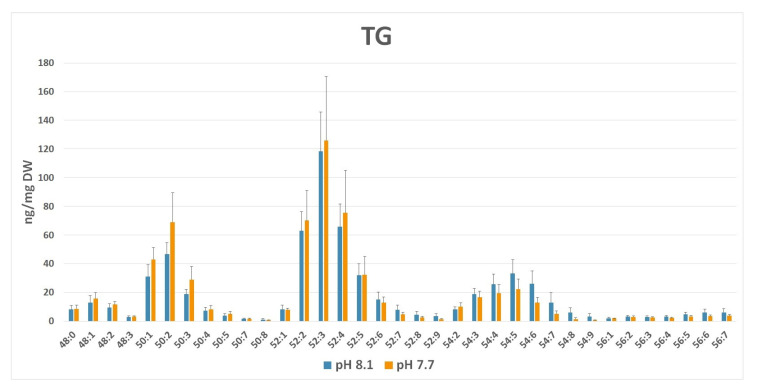
TG profile (ng/mg dry weight ± SD) in normal (pH 8.1) vs. acidic (pH 7.7) medium growth of *Ulva prolifera*. Lipid groups have been annotated as sum composition C:N (total number of carbons:total number of double bonds in fatty acid chains).

A closer inspection of the lipid species composition (Supplementary Figures S11–S13) indicated that other minor palmitoyl-based galactolipids, i.e., SQDG 16:0/16:0, DGDG 16:0/16:0, and MGDG 16:0/16:0, were slightly down-modulated, even despite the general trend of the corresponding classes, which exhibited an increased level at pH 7.7 (i.e., DGDG and MGDG). In TG, lipid species composition showed that TG group C52 was prominent in both conditions (Figure 10). This group corresponds to TG typically composed of one C16 and two C18 fatty acyl chains. The degree of unsaturation, i.e., the number of total double bonds present in each TG, regardless of the fatty acid nature, was indicated by the number, N (i.e., C52:N), after the group label. Indeed, at a lower pH (7.7), a shift in species abundance from TG C52–C56 towards TG C48–C50 groups bearing lower-length acyl chains was observed. In addition, a prevalence of saturated FAs bound to glycerol occurred, as attested by the predominance of the low number of double bonds (N) in annotated C48–C52 class groups. These results suggest an acidified medium-driven TG composition modulation with a lower occurrence of long polyunsaturated chain C18–C20 fatty acids in the neutral lipid pool.

## 3. Discussion

The decrease in seawater pH recorded in the last decades stimulated a great deal of research on the effects of OA on various organisms. Indeed, clear effects of OA on the composition of various plants have been demonstrated, inducing physiological effects on various consumers [52,53]. Previous studies indicated the effect of OA on the production of infochemicals by microalgae and seagrasses [5]. The NMR- and MS-based metabolomics and lipidomics are increasingly being applied in ecological studies to profile and quantify small molecules and lipids that represent the biological response of living organisms to changing environmental conditions as a direct consequence of genomic and transcriptomic activity [54,55]. Here, NMR metabolomics has been used to assess the changes in water-soluble metabolites, while LC-MS/MS approaches were applied to monitor medium-polarity molecules and lipids in algal extracts.

### 3.1. Pathway Analysis

Our NMR-based metabolomic analysis of *U. prolifera* under acidified conditions showed that the arginine, asparagine, glutamine, and glutamate levels increased along with the enhanced growth rate. At the same time, other metabolites such as choline, phosphocreatine, sucrose, and DMSP decreased. To shed light on the biochemical mechanisms underlying these metabolomic changes in *U. prolifera* in response to OA, we investigated the metabolic pathways in which the differentially modulated metabolites were involved. Under acidified conditions, arginine biosynthesis, alanine, aspartate and glutamate metabolism, arginine and proline metabolism, starch/sucrose metabolism, one-carbon pool by folate, sulfur metabolism, and ABC transporters were the most strongly affected pathways (Figure 4, pathways 1–8). Moreover, the analysis identified specific sub-pathways involved: urea cycle, spermidine, and putrescine polyamine biosynthesis from arginine, betaine biosynthesis from choline, dissimilatory sulfate reduction, sulfate–sulfur assimilation, arginine biosynthesis from ornithine, and glycogen degradation (Figure 4, sub-pathways 9–15).

### 3.2. Amino Acids

Seaweeds promptly respond to the altered environmental conditions by regulating their physiological and biochemical processes, mainly carbon and nitrogen metabolism, which leads to alterations in various metabolic networks linked to amino acids (AAs) and organic acids [23,30]. Here, we demonstrated that glucogenic amino acids such as glutamine and glutamic acid, together with arginine and asparagine, were accumulated at a lower pH, which may serve as precursors for diverse metabolic pathways and major central organic nitrogenous compounds involved in the storage and transport of nitrogen. It has been shown that the higher availability of dissolved CO_2_ facilitates the photosynthesis of algal species, which can capitalize on increased carbon availability, thus favoring their growth rates [27,56]. This goes along with enhanced protein biosynthesis, increasing the total protein and amino acids content in algal tissues [32,57,58]. Above all, arginine, glutamate, and aspartate are generally reported as the most abundant AAs in *Ulva* species [59,60,61]. Fundamental AAs, including asparagine and glutamine, may be a source of ammonia, useful to buffer external acidic conditions or conversely involved in nitrogen recycling [61] and algal detoxification [62]. As also observed in higher plants, these amino acids contribute to the tricarboxylic acid (TCA) cycle-related deamination and transamination reactions in macroalgae [63]. Moreover, glutamate is assembled in glutamate dehydrogenase (GDH), which plays an essential role in the regulation of ammonia assimilation in such species of algae as *Chlorella sorokiniana* [64], *Ulva pertusa* [65], *Chlamydomonas reinhardtii* [66], *Bryopsis maxima* [67], and *Pyropia yezoensis* [68]. Our results suggest that the increases of these AAs under acidified conditions might reflect the adjustment of some nitrogen metabolism pathways as algal responses to environmental stress. As indicated by the enrichment analysis (Figure 4), the urea cycle is one of the modules supposed to be altered under the acidification condition. In particular, this may reflect the high recycling rate for ammonia assimilation used to counteract the acid environment and the urea production, which is an internal organic source of nitrogen produced by the catabolism of arginine [69]. This is consistent with our experimental findings: at low pH values, we observed a higher content of asparagine, derived from aspartate via transamination in the urea cycle, also predicted to be involved from the pathway analysis as an additional metabolite. Aspartate, together with citrulline, are at the top of the urea cycle, both precursors of arginine—experimentally observed as upregulated—which in turn is transformed into ornithine, that again catabolizes citrulline via ornithine carbamoyltransferase, thus restarting the cycle and re-producing arginine. Actually, the arginine biosynthesis from ornithine was also predicted to be altered from our enriched analysis. Ammonia, the final form for nitrogen fixation, is also the readily available source for glutamine biosynthesis via glutamine synthetase and then glutamate, both found in our study highly expressed in acid conditions. Those metabolites contribute in catalyzing the entry of bioavailable nitrogen in the form of ammonium into the cellular metabolism, being alternatively involved in the glutamate dehydrogenase (GDH) and the glutamine synthetase/glutamate synthase (GS/GOGAT) cycle [70,71].

On the other hand, low pH regimes imply algal osmotic adaptation and a protective response to maintain intracellular pH, thus preserving normal metabolic processes and cellular functions. The algal response can be related to the consumption of choline, sucrose, and DMSP, which may act as osmolytes [72] and protein stabilizers [73]. Osmolytes are recruited and accumulated around the protein backbone to exert a stabilizing effect through polar interactions [73].

### 3.3. Carbohydrates

Carbohydrates are vital for algal growth and development, being the primary source of energy for respiration and other essential processes; in addition, soluble carbohydrates can serve as precursors of diverse bioactive metabolites and as building blocks for the synthesis of essential polymers, including starch, cellulose, and proteins, which increased at low pH levels [74]. Hence, their lower concentration observed in our results may be related to the higher demand for energy and the synthesis of primary metabolites in fast-growing algae under high pCO_2_. Moreover, sucrose metabolism provides a mechanism to reduce its concentration at the unloading sites, to facilitate its source-to-sink translocation, thus preventing feedback inhibition of photosynthesis and sustaining carbon flow at the whole-plant level [75,76]. Decreased sucrose levels in acidified conditions may be further related to an inverse regulation of sucrose transporters in plants and fungi, depending on extracellular pH [77]. Thus, its depletion at pH 7.7 is related to its use to counteract acidification [78,79,80].

The compound **145**, analyzed by an LC-MS-based approach, was putatively identified as (2*R*)-3,4,5-Trisulfooxyoxane-2-carboxylic acid, which is a highly sulfated deoxyglucuronic acid (Figure 7A). Glucuronic acid is a basic unit of ulvans, cell wall polysaccharides abundant in green seaweed of the genus *Ulva* [81,82,83,84]. Therefore, compound **145** derived from glucuronic acid may represent a potential chemical biomarker for algal biomass increase.

### 3.4. Other Small Polar Metabolites

Choline is a source of methyl groups needed for many metabolic steps. Its employment is related to a demanding request for enhanced methylation, occurring in all biochemical processes (accompanying a higher growth rate) involved in the production of proteins and enzymes. Choline is also a precursor for the synthesis of phosphatidylcholines, phospholipids vital for cell structure and signaling. Its reduction in favor of cell membrane constituents (in line with the PC increase found in MS analysis) could be related to membrane adaptation to acidification, which can induce lipid vesicle migration and global deformation [85]. The control of membrane lipid composition and its rearrangement to preserve integrity and fluidity is fundamental in maintaining cytoplasmic pH, which is essential for optimal cellular metabolism, growth, and proliferation [86]. Moreover, membrane participation in pH homeostasis involves regulating transmembrane trafficking and proton transporters, which enhance the pumping of H^+^ ions in acidic environments [87]. In addition, the choline decrease may be related to its metabolization into dimethylglycine (DMG), whose signal resonates overlapping the S-CH_3_ peak of DMSP. Indeed, the bin levels were higher for the pH 7.7 group than for the controls, although not significant in Student’s *t*-test (data not shown). DMG is an essential osmoregulatory metabolite that may counteract cell wall expansion and osmotic pressure at a low pH [88]. Choline is also the precursor of S-adenosylmethionine, a universal methyl donor involved in synthesizing homocysteine in sulfur metabolism, and methionine, whose mean levels were upmodulated in the pH 7.7 group compared to controls, although not significant in the Student’s *t*-test (data not shown).

Phosphocreatine is a high-energy organic phosphate compound naturally occurring in organisms, playing a pivotal role in cellular energy metabolism as an immediately available temporal energy buffer and a spatial energy buffer or intracellular energy transport system [89]. Besides its prominent role in coping with the increased energy demand realized under experimental conditions, phosphocreatine is also involved in the algal response to the acid environment by an extensive generation of ATP. Metabolic processes such as creatine phosphate hydrolysis, ATP formation, or consumption of CO_2_ imply a net consumption of H^+^, thus contributing to the cellular alkalinizing process [90]. It has been shown in the microalga *C. reinhardtii* that 7% more ATP was consumed to remove protons entering the cytosol across the membrane during growth at a lower pH [91,92]. Thus, lower levels of phosphocreatine found in acidified conditions may be related to a considerable increase in ATP and photophosphorylation demand and an impaired turnover.

### 3.5. Sterols

A significant increase upon acidification was observed in the concentration of compound **66**, identified as 24-methylene-cycloartanol (Figure 7C), an essential precursor in the biosynthesis of sterols in photosynthetic lineages [93]. Sterols are fundamental constituents of membranes, playing an essential role in the lipid bilayer’s integrity, fluidity, and permeability, and are involved in hormone signaling [94,95]. In plants, sterols can be synthesized by two pathways: via lanosterol, as in mammals and yeast, or cycloartenol metabolism [96]. In the latter pathway, cycloartenol is converted into 24-methylene-cycloartanol (compound **66**) by the catalysis of the cycloartenol-C-24-methyltransferase 1, an enzyme that has already been found upregulated in *U. prolifera* under environmental stress conditions [97]. The up-modulation of 24-methylene-cycloartanol under acidified conditions might indicate an increment in the biosynthesis of phytosterols via cycloartenol. This putative increase in phytosterol levels could be a mechanism to regulate membrane fluidity and acclimate the tissues to an acidified environment [98] or just the demand for new membrane components and signals due to the increase in biomass. This up-modulation is also in agreement with the observed increase of ABC transporters.

### 3.6. Signaling and Deterrent Compounds

DMSP is a tertiary sulfonium compound massively produced by several species of marine micro- and macro-algae and plant halophytes, including Ulvaceae [99], and it is considered to have osmotic, cryoprotectant, chemoattractant, and antioxidant roles [72,100,101]. Via enzymatic cleavage, DMSP may serve as a precursor of DMS (dimethyl-sulfide), a gas whose emission influences atmospheric processes and climate. For this reason, most previous studies focused on the importance of both compounds (DMSP and DMS) and their oceanic planktonic sources to global sulfur cycling and climate [102]. DMSP mediates ecological interactions between the producing organisms and the surrounding marine community, and this observation boosted renewed interest in this metabolite [103]. Interestingly, DMSP may be involved in food deterrence by producing DMS and other by-products, representing an activated chemical defense against herbivores of other sympatric algae [103]. However, the multiple roles attributed to DMSP are species-specific and modulated by adaptation to local environmental conditions. Several studies focused on the effects of OA on the algal production of both DMSP and its enzymatic products. Indeed, contradictory results reported variable responses of DMSP and DMS to OA, with positive, negative, or no correlation between the two metabolites [104,105,106,107,108]. The reduction of DMSP observed in our study may be the result of its imbalanced conversion into DMS in a short timescale or, in contrast, an indicator of higher exposure of the alga to grazing pressure. Two putative macrolide derivatives were up-modulated by the acidification (compounds **9** and **151**, respectively, in Figure 7B,E). These compounds’ class has been previously identified in algae [109] and is abundant in various marine organisms [110]. Moreover, macrolides have displayed vigorous deterrent activity in laboratory feeding assays against fish [111,112]. Consequently, under acidification stress, these defense metabolites would contrast grazing pressure. This observation is in line to what was previously indicated by Gaubert et al. [49]. In fact, they investigated a brown macroalga and recorded a significant decrease in some specialized metabolites’ concentrations at a lower pH, including lobophorenols B and C and other oxylipin derivatives, concluding that the downregulation of metabolic pathways involving lobophorenols is in accordance with the optimal defense theory.

### 3.7. Lipids

The lipid pool, widely distributed in several classes, constitutes structural or storage material in all living cells. As already documented for other *Ulva* species [56], we registered a significant increase in lipid yield in samples grown in acidic conditions, in line with the increment of algal productivity observed (Figure 1 and Supplementary Figure S7). However, no statistical significance was detected at the level of specific classes or individual lipid species. This may be due to the variability among biological replicates in both pH conditions, which was particularly amplified in the lipidome with respect to the polar metabolome. Nevertheless, we attempted to seek trends, focusing on the mean values of class lipids and individual molecular species. As a result of the fold-change analysis, a series of lipid classes appeared upmodulated, mainly belonging to phospholipids (PL), including PC, PE, and PG (Supplementary Figures S8–S10). PL are sensitive to various stresses, including temperature, light, nutrients, and pH. PG are the most abundant phospholipids occurring in chloroplasts. Generally, PG decreases under various stress conditions, particularly in acidified environments [113]. On the other hand, recent reports described a significant (up to 80%) upregulation of PG under long-term high CO_2_ [114,115]. The medium/long-term adaptation may favor this class of anionic lipids negatively correlated to SQDG, other anionic plastidial lipids [116]. Indeed, our experiments indicated that the general increase of PG is balanced by a decrease of sulfoquinovosides, particularly evident for SQDG 16:0/16:0, putatively as a strategy to face the lower pH and maintain a balanced negative environment (Supplementary Figure S13); however, the molecular basis of this mechanism is understudied in photosynthetic organisms [115].

PE and PC are both constituents of cell membranes: the PE class is a lipid chaperone that assists in the folding of specific membrane proteins and is required for the activity of several of the respiratory complexes, while PC also play a role in membrane-mediated cell signaling. In *U. prolifera,* they were composed mainly of saturated and monounsaturated fatty acids, and under high CO_2_ they exhibited a general increase, likely as a consequence of the activated metabolism in this condition. PI belong to the pool of anionic PL important as membrane components and lipid mediators, particularly in deacylated or phosphorylated forms, modulating a series of growth-related physiological processes. Most of the studies reported for this class of lipids concern mammalian cells, focusing on PI 18:0/20:4 and terrestrial plants. However, accumulating evidence extends the occurrence of the PI signaling pathway to micro- and macro-algae [117]. Although, PI class profiling showed only the occurrence of C16- and C18-based PI lipids. In our experimental conditions, their level did not change on average with the lowered pH, except for PI 16:0/16:0, which was reduced (Supplementary Figure S14). This may suggest a specific regulation or structural role for this lipid composed of two residues of palmitic acid.

Other lipid species, such as SQDG 16:0/16:0 and DGDG 16:0/16:0, composed exclusively of palmitic acid, showed a singular behavior, resulting in a reduced content, while the other members of the corresponding class increased or were unchanged (Supplementary Figures S11–S13). Conversely, we observed an apparent increase in TG species containing palmitic acid (TG 48–52). This increment was accompanied by a general reduction of TG species containing PUFAs (TG 54–56) (Figure 9 and Figure 10). It has been reported in the red alga *Galdiera sulphularia* [113] and the green alga *Chlamydomonas* sp. [118] that the composition of fatty acids in the various classes of lipids depends on the pH of the medium and the content of palmitic acid in TG increases when the ambient pH decreases. On the other hand, decreasing pH produces a decline in the proportion of PUFA at the base of the marine food webs, i.e., in picoeukaryotes [119] and diatoms [115,120], with a bottom-up impairment in the transfer of essential PUFA to higher levels [114]. A recent study on the brown macroalga *Sargassum vulgare* [121] also reported a reduction in PUFA, although in this case not accompanied by an increment of palmitic acid in the total fatty acid composition. However, the mechanism underlying this change is still unclear. High CO_2_ could likely stimulate biosynthesis and the accumulation of saturated FA and/or conversely may induce a downregulation of fatty acid desaturases [115,122]. This trend is important because it may produce predictable changes in the composition of algae, their nutritional value, and the production of lipophilic infochemicals. On the other hand, the remodeling associated with changes in the relative abundance of palmitic acid in various lipid classes and the possible intercorrelation among polar and neutral C16:0-based lipids deserve further investigation.

## 4. Materials and Methods

### 4.1. Collection and Isolation of the Macroalga

Algal thalli of *U. prolifera* were collected in Lacco Ameno (Ischia, Naples) [123] on the leaves of *P. oceanica* growing in a shallow stand (5 m depth) and morphologically identified as *Ulva prolifera* O.F. Müller according to Cui et al. [124] and transported to the laboratory for inspection under a stereomicroscope. Algae and adherent materials’ samples were transferred to multi-wells containing 6 mL of Guillard’s f/2 medium [125]. Multi-wells were kept in a thermostatic chamber (18 °C, photoperiod 12/12 h) for 1–2 days. Algal thalli were then re-collected and transferred to new wells containing sterile f/2 medium several times until a pure culture of *U. prolifera* was obtained. The absence of algal or bacterial contaminants was confirmed through optical microscopy observations. The mother culture was kept in a thermostatic chamber in the same medium and periodically transferred while checking the strain’s purity and physiologic state.

### 4.2. Production of Ulva Biomass in Benthic Photobioreactors

Special photobioreactors were ad hoc designed to produce macroalgae at normal pH and acidified conditions. To this end, plastic tanks with a total volume of 5 L were sanitized by washing with H_2_O_2_ and then rinsed with Milli-Q water before filling with sterilized f/2 medium. Each photobioreactor contained a pH probe and a “U”-shaped glass pipette connected with a CO_2_ bottle whose valve was operated by an automatic controller (CO_2_ controller, Aquauno, Lecce, Italy). Four tanks (biological replicates) were set at pH 8.1 and four additional tanks were set at pH 7.7.

**Figure 11:**
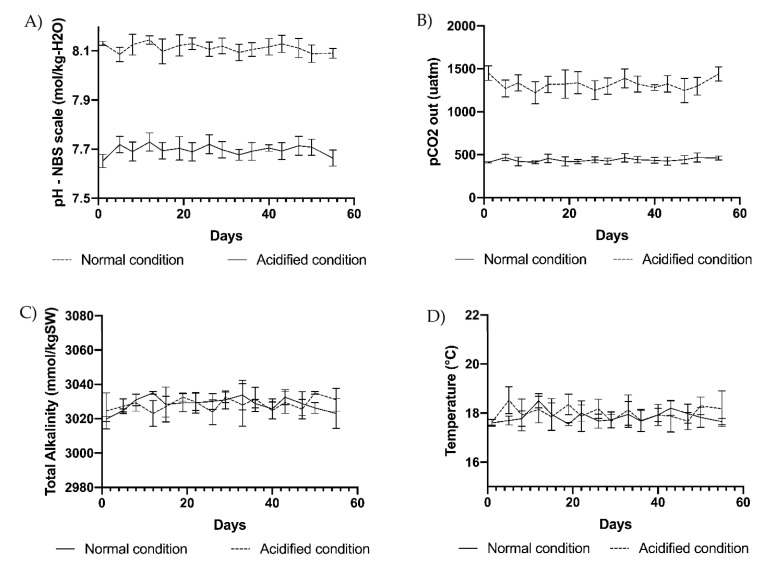
(**A**) pH, (**B**) pCO_2_, (**C**) total alkalinity, and (**D**) temperature measured at 3-day intervals in the 4 replicate tanks at ambient pH of 8.1 (normal condition) and in 4 replicate tanks at acidified pH of 7.7 (acidified condition). Vertical bars indicate SD on means of 4 biological replicates.

The photobioreactors were built to react to any pH change due to gas exchanges over the water surface and to the effects of photosynthesis and respiration processes in each tank. Consequently, pH changes were immediately corrected by small additions of CO_2_ up to reaching the pH initially set on the controller. Tanks were covered with a glass lid and kept in a thermostatic chamber at a temperature of 18 °C (photoperiod 12/12 h, light irradiance of about 350 µE provided by Sylvania Gro Lux neon lamps). A thallus of *U. prolifera* (3 g fresh weight) was added to each of the 8 tanks initially kept at ambient pH (8.1), and the pH of the tanks was slowly adjusted up to the final sets (pH 8.1 and pH 7.7) for 2 days prior to the start of the experiment. The mopped fresh weight of each thallus was further recorded twice a week for 55 days. In each replicate, 50 mL samples of culture medium were collected every 3 days to perform analyses of the carbonate chemistry. Samples were analyzed immediately after the collection in the photobioreactors, avoiding the formation of air bubbles possibly inducing impairment of the measure. The controller continuously checked the pH which was additionally recorded every 3 days using a SevenGo (Mettler Toledo, Milan, Italy) pH probe (Figure 11A). At the same time intervals, the temperature was checked using a digital thermometer (Figure 11D) and total alkalinity (TA) was measured using the total alkalinity mini titrator for water analysis HI-84531-02 (Hanna Instruments, Woonsocket, RI, USA) (Figure 11C). The instrument automatically adjusted the working temperature (18 °C) using the automatic temperature compensation feature. Three points of electrode calibration (pH 4.01, 7.01, and 8.30 at 25 °C) and pump calibration (using a HI 84531–55 calibration standard) were performed before each set of titrations to ensure accuracy. The corresponding partial pressure of CO_2_ (pCO_2_) was computed every 3 days using the CO_2_Sys EXCEL Macro from pH NBS, TA, coupled with temperature and salinity records [126]. Carbonic acid dissociation constants (pK1 and pK2), ion HSO_4_^−^, and borate dissociation constants were used for the computation (Figure 11B). Salinity was periodically measured with a HI-96822 (Hanna Instruments, Woonsocket, RI, USA) electronic refractometer.

At the end of the experiment, the biomass obtained in each of 4 biological replicates for the 2 conditions was collected, weighed (fresh weight), and frozen for the chemical analyses. The collected algae were checked under optical microscopy to confirm the absence of evident bacterial growth: no traces of bacterial contamination were detected in any of the replicates submitted to further analyses. The weight data collected during the experiment were used to build growth curves (according to a logistic equation, Supplementary Method S1) for *U. prolifera* in normal and acidified conditions.

### 4.3. NMR Metabolomics

#### 4.3.1. Sample Extraction and NMR Analysis

Frozen algal samples were divided into four portions of about 1 g each to obtain four technical replicates for the two experimental conditions (pH 8.1—control vs. pH 7.7—treatment). Briefly, 170 μL of water and 700 μL of methanol (MeOH) were added, and the samples were sonicated for 30 s. Then, 350 μL of chloroform was added, and after vortexing and shaking for 10 min, 350 μL of water and 350 μL of CHCl_3_ were added. After centrifugation at 10,000 g for 10 min at 4 °C, the upper phase containing polar metabolites was collected, lyophilized, and stored at −80 °C until analysis.

NMR analyses were performed on a Bruker Avance III–600 MHz spectrometer with an autosampler (Bruker BioSpin GmbH, Rheinstetten, Germany), equipped with a TCI CryoProbeTM fitted with a gradient along the *Z*-axis, at a probe temperature of 27 °C. Details for 1D and 2D experiments [127,128,129,130,131,132] are reported in the Supplementary Materials. Metabolites were assigned to NMR profiles by comparison of chemical signal shifts (^1^H and ^13^C nuclei) with literature data [133] and/or an online database [134].

#### 4.3.2. Multivariate Data Analysis

For NMR data, the 0.70–8.70 ppm spectral region of aqueous extracts was automatically reduced to integrated regions (buckets) of a 0.02 ppm width using the AMIX 3.9.7 package (Bruker Biospin GmbH, Rheinstetten, Germany). The residual water resonance region (4.50–5.18 ppm) in ^1^H_2_O spectra was excluded. Each integrated region was normalized to the total spectrum area to avoid possible signal variation due to the dilution of samples. The integrated data organized in matrices format were then imported into the SIMCA14 package (Umetrics, Umeå, Sweden), where principal component analysis (PCA) and orthogonal projection to latent structures (OPLS) or its discriminant analysis (OPLS-DA) were performed. Pareto scaling was used as data pre-treatment for PCA and OPLS-DA in NMR data. PCA was first applied (data not shown) as an unsupervised strategy to display the dataset variation of the data matrix to identify data trends. However, an OPLS-DA was performed to visualize data clustering to the different pH growth conditions. The discriminatory metabolites were selected based on the bins containing NMR signals which were not overlapping with other peaks for the relative quantification, performed using the OriginPro 9.1 software package (OriginLab Corporation, Northampton, PA, USA). Statistical significance for selected metabolites was determined by parametric (Student’s *t*-test) or non-parametric (Mann–Whitney U) tests according to the results of the normality test performed on data to evaluate each distribution (Shapiro–Wilk and Kolmogorov–Smirnov test, together with Levene’s test to evaluate variance homogeneity). *p*-values < 0.05 were considered statistically significant.

#### 4.3.3. Enrichment Analysis

Enrichment analysis on the most representative metabolites found in *U. prolifera* cultured in acidification conditions was applied using the ‘diffusion’ method computed by the FELLA package in R [135,136]. Such analysis suggests affected reactions, enzymes, modules, and pathways using label propagation in a knowledge model network based on the green alga *Chlamydomonas reinhardtii* database in KEGG. The resulting network and sub-network are visualized in a related plot and table with a threshold of *p* < 0.05.

### 4.4. LC-MS Metabolomics

#### 4.4.1. Sample Extraction and LC-MS Analysis

Dried samples (10 mg) from algal specimens grown at normal (four replicates) and acidified (three replicates) conditions were ground separately for 30 s at 5000 rpm in a tissue homogenizer (Minilys, Bertin Technologies, Germany) by using six zirconium oxide beads (1.4 mm, Bertin Technologies, Germany). Then, 200 µL of prechilled Milli-Q water was added and vortexed for 30 s, then sonicated for 1 min. After 30 min, 800 µL of acetonitrile (ACN) (LC-MS grade) was added, and the samples were vortexed again for 30 s. Samples were then stored overnight at 4 °C to facilitate the extraction of metabolites and then vortexed and centrifuged at 4000× *g* for 10 min at 4 °C. Supernatants (800 µL) were then transferred separately into 1.5 mL glass vials and dried in a vacuum concentrator (RVC 2–25, Christ, Germany) at 40 °C. Samples were dissolved in 300 µL of 100% acetonitrile (LC-MS grade) and kept at −20 °C until LC-MS analysis. Equal aliquots from all samples were pooled and used as a quality control (QC) sample.

The metabolic profiles were acquired by injecting 10 µL of each sample into a UHPLC-Q Exactive HF Orbitrap System (Thermo Scientific, San Jose, CA, USA). The separation of the metabolites was achieved in a Vanquish Horizon Binary UHPLC system at a constant flow rate of 400 µL min^−1^ on a C18 reversed-phase column (EC 125/2 Nucleosil 100-3 C18, Macherey-Nagel, Germany), using a solvent gradient of 0.1% (*v*/*v*) formic acid (FA) in water (solvent A) and 0.1% FA in ACN (solvent B) as follows: 0–0.3 min isocratic 80% A, 0.3–7 min gradient phase to 100% B, 7–11 min isocratic 100% B, 11.0–11.1 min gradient to 80% A, and 11.1–13 min isocratic 80% A. The samples were measured in negative ionization mode with a resolving power of 120,000 m/Δm and a mass range of 75–1125 *m*/*z*. The electrospray ionization (ESI) source was set at a 2.5 kV spray voltage, 35 V for capillary transfer voltage, and a capillary temperature of 360 °C.

Full MS scan and data-dependent MS2 (Full MS/dd-MS2 (top N)) acquisitions were performed on the QC sample. The injection volume, chromatographic method, and instrumentation were the same as described in the previous paragraph. For the Full MS acquisition, a resolution of 120000 m/Δm, automated gain control (AGC target) set to 3 × 106, the scan range of 75–1125 *m*/*z*, and a maximum injection (IT) time of 100 ms were used. The parallel dd-MS2 (top N) was acquired for the top 20 most intense *m*/*z* by using a resolution of 120000 m/Δm, an AGC target of 1 × 105, maximum IT of 100 ms, isolation window of 1.0 *m*/*z*, and normalized collision energies of 15, 30, and 45 eV.

#### 4.4.2. LC-MS Data Processing

The raw data files obtained from the LC-Orbitrap MS system were initially converted to mzXML format using the MSconvert tool from ProteoWizard 3.0x software [137], and mzXML files of all replicates were processed with the XCMS 3.2.0 R package [138,139,140]. Peak picking was performed with the centWave algorithm (ppm = 1, peakwidth = c(5, 30), prefilter = c(6, 50,000), mzCenterFun = “wMean”, integrate = 1, mzdiff = –0.001, fitgauss = TRUE, noise = 1E4, verboseColumns = TRUE). An initial alignment of the selected peaks was carried out (bw = 2, mzwid = 0.009, max = 100, minsamp = 0, sleep = 0). Then, a retention time correction was performed using the obiwarp method, followed by a second peak alignment (bw = 2, mzwid = 0.009, max = 100, minfrac = 0.5, minsamp = 1, sleep = 0). All masses detected in the blank, and those with coefficients of variation for the intensities higher than 20% in the QC sample, were removed from the mass peak list. Finally, the CAMERA 1.36.0 R package [141] annotated possible isotopes and adducts in the peak matrix and generated the final peak intensities list for further statistical analysis.

#### 4.4.3. Statistical Analysis

Multivariate and univariate statistical analyses were performed to reveal the relationships between the metabolic profiles of *U. prolifera* cultivated at pH 7.7 vs. those at pH 8.1 using the MetaboAnalystR 2.0 package [142]. The peak intensities table was initially normalized by sample-specific weights and later log-transformed and scaled by mean-centering and dividing by the square root of the standard deviation of each variable (equivalent to Pareto scaling). To visualize changes in the metabolic profiles at pH 7.7 compared to 8.1, a partial least square discriminant analysis (PLS-DA) was carried out. A volcano plot comparing pH 7.7/8.1 was executed (FC ≥ 2.0, *p*-value ≤ 0.05).

#### 4.4.4. Compound Identification

Only metabolites that were significantly modulated under acidified conditions (pH 7.7) compared to normal conditions (pH 8.1) with an FC ≥ 2.0 at *p* ≤ 0.05 from the volcano plot were putatively identified by initially calculating their exact masses from their accurate masses and the ion annotation information provided by CAMERA (adducts, isotopes, and neutral-losses fragments). Then, an isotopic pattern analysis and a library spectral matching, always allowing a mass deviation of 5 ppm in databases such as MetFrag, METLIN, SIRIUS, CSI: FingerID, Human Metabolome Database (HMDB), KEGG-mine, and LipidMaps, were performed to achieve a Level 2 (High) of annotation for all metabolites, except compound **103**, which remains unknown. The IUPAC name of compound **151** (Figure 7) was generated by using STOUT (SMILES-TO-IUPAC name translator) [143].

### 4.5. LC-MS Lipidomics

#### 4.5.1. Sample Extraction and LC-MS Analysis

Dried material (15 mg) from four biological replicates for each experimental condition (pH 8.1—normal vs. pH 7.7—treatment) was extracted in technical duplicates. A pool of lipid standards, commercial (C17-Triacylglycerol (TG), d5-C16-Diacylglycerol (DG), C10-Phosphatidylcholine (PC), C17-LysoPC, and C17-Phosphatidylethanolamine (PE), purchased from Avanti Polar Lipids) and synthetic (C19-Sulfoquinovosyldiacylglycerol (SQDG) and C19-Monogalactosyldiacylglycerol (MGDG)), was used for recovery assessment and relative quantification of metabolites [144]. Lipid extraction was performed according to the modified methyl-t-butyl-ether (MTBE) protocol [144,145]. Briefly, the dry material was suspended with 900 µL of LC-MS-grade MeOH and the pool of standards (final concentration between 0.2 and 2 μg/mL) was added. After vortexing, 3 mL of HPLC-grade MTBE was added, the samples were sonicated for 2 min, and then left at room temperature under shaking for 1 h. After adding 750 µL of MilliQ water and shaking for 10 min, the samples were centrifuged at 10,000× *g* for 10 min at 4° C, and the upper organic phase was recovered. The aqueous phase was extracted again with 1 mL of MTBE. The organic phases were combined, dried under a nitrogen stream, weighted, and stored at −80 °C until analysis. Lipid extracts were normalized to dry algal mass for samples grown in both conditions, and values were reported as percentage ± SE (Supplementary Figure S6).

For lipidome LC-MS analyses, each extract was reconstituted in 200 μL of MeOH/isopropanol (9:1) and analyzed by UHPLC-MS according to the method reported in [146]. Briefly, the chromatographic separation was achieved on an Infinity 1290 UHPLC System (Agilent Technologies, Santa Clara, CA, USA), equipped with a Kinetex Biphenyl column, 2.6 μm, 150 mm × 2.1 mm (Phenomenex, Castel Maggiore, Bologna, Italy), at 28 °C by using as eluent A: water/ACN 60:40, 10 mM HCOONH_4_, and 0.1% FA, and eluent B: isopropanol/ACN 90:10, 2 mM HCOONH_4_, and 0.1% FA. MS analyses were carried out on a Q-Exactive Hybrid Quadrupole-Orbitrap mass spectrometer (Thermo Scientific, San Jose, CA, USA) in both polarity modes in the mass range *m/z* 150–1800.

#### 4.5.2. Lipid Identification

Raw data from UHPLC-Q Exactive MS/MS analysis were directly processed by LipidSearch Software (Thermo Scientific, San Jose, CA, USA) with 5 ppm tolerance for the precursor ion and 10 ppm for the product ion. The m-score threshold was set to 5. The lipid identification lists were aligned and compared by their lipid class and lipid species levels using a retention time tolerance of ±0.25 min. The main grade was set to A, B, and C for all lipid classes. After peak alignment, the software yielded an output of 846 lipid ions, corresponding to: 168 TG, 22 DG, 4 monoacylglycerol (MG), 38 PC, 50 PE, 48 phosphatidylglycerol (PG), 16 phosphatidylinositol (PI), 7 phosphatidylserine (PS), 7 LysoPC, 3 LysoPE, 6 LysoPG, 1 LysoPI, 78 SQDG, 59 MGDG, 43 digalactosyldiacylglycerol (DGDG), 18 sulfoquinovosylmonoacylglycerol (SQMG), 5 monogalactosylmonoacylglycerol (MGMG), 4 digalactosylmonoacylglycerol (DGMG), 82 ceramide (Cer), and 17 glucosylceramide (GluCer) main molecular species. The primary adduct ion was set to H^+^ for PC, PE, LysoPC, and LysoPE, and to NH_4_^+^ for DG and TG. Cer, GluCer, SQDG, SQMG, MGDG, MGMG, DGDG, and DGMG were measured as HCOO^-^ adducts, while PI, PS, and PG were monitored as deprotonated ions. Manual filtering accounted only for species with confirmed lipid annotation at the FA level (Level 2, High). Regiochemistry was not assigned. Further, lipids were quantified by normalizing to the specific IS for each class, and a second filtering step was applied mainly to TG to filter out less represented components.

#### 4.5.3. Statistical Analyses

Statistical analyses for MS-metabolomics were described above. To identify lipids that have been modulated under acidified conditions (pH 7.7) as compared to normal conditions (pH 8.1), a fold-change analysis comparing pH 7.7/8.1 was executed (FC ≥ 2.0). Volcano plots were obtained to facilitate the discrimination of changes in compound composition. The significance of differences between the final weights of algae obtained under different pH conditions was evaluated by the Student’s *t*-test. A Mann–Whitney test was performed to evaluate the differences in the growth curves under different pH levels. The slopes of the growth curves obtained for normal and acidified conditions were evaluated according to confidence intervals. The changes in the algal composition profiles in response to acidification were evaluated by one-way ANOVA. All tests were performed using GraphPad Prism ver. 8 for Mac (GraphPad Software, San Diego, CA, USA).

## 5. Conclusions

The outcome of our research study reinforces the use of common and ubiquitous green algae, such as *Ulva prolifera*, considered to be excellent models for investigating the effects of OA on green algae. We revealed that *U. prolifera* grows at higher rates in acidified environments than ambient pH conditions. Besides, as a result of applying complementary LC-MS- and NMR-based untargeted metabolomics, changes in various classes of metabolites were revealed, providing essential information about the most impacted metabolic pathways in *U. prolifera* by OA. The observed significant increases in the amino acids by OA likely reflect the adjustment of nitrogen metabolism pathways. Combined with the modulation of carbohydrates, *U. prolifera* may preserve normal metabolic processes and cellular functions from changing pH conditions. A variation in the level of metabolites such as a sulfated glucuronic acid derivative, 24-methylene-cycloartanol, and choline, which are implicated in the biosynthetic pathways of essential macromolecules in green algae, might be associated with their demand by the high growth rates observed under acidified conditions or by the structural adaptation to the changing environment. Similarly, phosphocreatine might be modulated as a response to the energy demanded by the increased growth or to cope with the H^+^ increase at low pH conditions. OA also modulated some important deterrent compounds. DMSP might have been consumed to synthesize DMS, that in conjunction with the increase of two putatively identified macrolides, may protect the alga more from grazing under stress conditions. In addition, a remarkable shift was observed here for the first time in the fatty acid composition of triglycerides and individual levels of phospholipids and glycolipids. These variations mainly affect PUFAs’ content and C16-based lipid species. Besides their biochemical significance and possible effects on animal consumers, these lipid changes should be considered for all the potential applications of these algae since they drastically modify their nutritional and nutraceutical value [147]. The reported results are likely to be further investigated and extended to other species of algae and should be considered for the forecast of indirect effects of OA on the food webs of marine benthic communities.

## Data Availability

The data presented in this study are available on request from the corresponding authors.

## Supplementary Materials

The following supporting information can be downloaded at: https://www.mdpi.com/article/10.3390/md20120743/s1, Table S1: Parameters of the logistic growth evaluated for Ulva prolifera cultivated at two pH regimes. Table S2: Ulva prolifera enrichment analysis. Figure S1: MS2 spectra of m/z 452.8875, [M + Cl]− adduct of compound 145. Figure S2: MS2 spectra of m/z 695.3517, [M − 2H + Na]− adduct of compound 9. Figure S3: MS2 spectra of m/z 475.3714, [M + Cl]− adduct of compound 66. Figure S4: MS2 spectra of m/z 617.3595 (unknown compound 103). Figure S5: MS2 spectra of m/z 528.3198, [M − H]− ion of compound 151. Figure S6: Lipid yield after Ulva prolifera extraction by MTBE/MeOH. Figure S7: Class profile lipidome of Ulva prolifera grown in normal (pH 8.1, replicates c, s1–s3) vs. acidic (pH 7.7, replicates s4–s7) conditions. Figure S8: Phosphatidylcholine (PC) composition and semi-quantitation in Ulva prolifera grown at pH 8.1 and pH 7.7. Figure S9: Phosphatidylglycerol (PG) composition and semi-quantitation in Ulva prolifera grown at pH 8.1 and pH 7.7. Figure S10: Phosphatidylethanolamine (PE) composition and semi-quantitation in Ulva prolifera grown at pH 8.1 and pH 7.7. Figure S11: Monogalactosyldiacylglycerol (MGDG) composition and semi-quantitation in Ulva prolifera grown at pH 8.1 and pH 7.7. Figure S12: Digalactosyldiacylglycerol (DGDG) composition and semi-quantitation in Ulva prolifera grown at pH 8.1 and pH 7.7. Figure S13: Sulfoquinovosyldiacylglycerol (SQDG) composition and semi-quantitation in Ulva prolifera grown at pH 8.1 and pH 7.7. Figure S14: Phosphatidylinositol (PI) composition and semi-quantitation in Ulva prolifera grown at pH 8.1 and pH 7.7. Methods S1: Growth curves’ logistic equation. Methods S2: NMR spectra acquisition and parameters.

## References

[1] Feely R.A., Sabine C.L., Lee K., Berelson W., Kleypas J., Fabry V.J., Millero F.J. (2004). Impact of Anthropogenic CO_2_ on the CaCO_3_ System in the Oceans. Science.

[2] Kroeker K.J., Kordas R.L., Crim R.N., Singh G.G. (2010). Meta-Analysis Reveals Negative yet Variable Effects of Ocean Acidification on Marine Organisms. Ecol. Lett..

[3] Doney S.C., Fabry V.J., Feely R.A., Kleypas J.A. (2009). Ocean Acidification: The other CO_2_ Problem. Ann. Rev. Mar. Sci..

[4] Friedlingstein P., Jones M.W., O’Sullivan M., Andrew R.M., Bakker D.C.E., Hauck J., Le Quéré C., Peters G.P., Peters W., Pongratz J. (2022). Global Carbon Budget 2021. Earth Syst. Sci. Data.

[5] Zupo V., Maibam C., Buia M.C., Gambi M.C., Patti F.P., Scipione M.B., Lorenti M., Fink P. (2015). Chemoreception of the Seagrass Posidonia Oceanica by Benthic Invertebrates Is Altered by Seawater Acidification. J. Chem. Ecol..

[6] Koch M., Bowes G., Ross C., Zhang X.H. (2013). Climate Change and Ocean Acidification Effects on Seagrasses and Marine Macroalgae. Glob. Chang. Biol..

[7] Manzello D.P. (2010). Coral Growth with Thermal Stress and Ocean Acidification: Lessons from the Eastern Tropical Pacific. Coral Reefs.

[8] Hendriks I.E., Duarte C.M., Álvarez M. (2010). Vulnerability of Marine Biodiversity to Ocean Acidification: A Meta-Analysis. Estuar. Coast. Shelf Sci..

[9] Atkinson M.J., Cuet P. (2008). Possible Effects of Ocean Acidification on Coral Reef Biogeochemistry: Topics for Research. Mar. Ecol. Prog. Ser..

[10] Orr J.C., Fabry V.J., Aumont O., Bopp L., Doney S.C., Feely R.A., Gnanadesikan A., Gruber N., Ishida A., Joos F. (2005). Anthropogenic Ocean Acidification over the Twenty-First Century and Its Impact on Calcifying Organisms. Nature.

[11] Hall-Spencer J.M., Rodolfo-Metalpa R., Martin S., Ransome E., Fine M., Turner S.M., Rowley S.J., Tedesco D., Buia M.C. (2008). Volcanic Carbon Dioxide Vents Show Ecosystem Effects of Ocean Acidification. Nature.

[12] Langdon C., Atkinson M.J. (2005). Effect of Elevated PCO_2_ on Photosynthesis and Calcification of Corals and Interactions with Seasonal Change in Temperature/Irradiance and Nutrient Enrichment. J. Geophys. Res. Ocean..

[13] Scartazza A., Moscatello S., Gavrichkova O., Buia M.C., Lauteri M., Battistelli A., Lorenti M., Garrard S.L., Calfapietra C., Brugnoli E. (2017). Carbon and Nitrogen Allocation Strategy in Posidonia Oceanica Is Altered by Seawater Acidification. Sci. Total Environ..

[14] Listiawati V., Kurihara H. (2021). Ocean Warming and Acidification Modify Top-down and Bottom-up Control in a Tropical Seagrass Ecosystem. Sci. Rep..

[15] Reiskind J.B., Seamon P.T., Bowes G. (1988). Alternative Methods of Photosynthetic Carbon Assimilation in Marine Macroalgae. Plant Physiol..

[16] Key R.M., Kozyr A., Sabine C.L., Lee K., Wanninkhof R., Bullister J.L., Feely R.A., Millero F.J., Mordy C., Peng T.H. (2004). A Global Ocean Carbon Climatology: Results from Global Data Analysis Project (GLODAP). Glob. Biogeochem. Cycles.

[17] Garrard S.L., Gambi M.C., Scipione M.B., Patti F.P., Lorenti M., Zupo V., Paterson D.M., Buia M.C. (2014). Indirect Effects May Buffer Negative Responses of Seagrass Invertebrate Communities to Ocean Acidification. J. Exp. Mar. Bio. Ecol..

[18] Hale R., Calosi P., Mcneill L., Mieszkowska N., Widdicombe S. (2011). Predicted Levels of Future Ocean Acidification and Temperature Rise Could Alter Community Structure and Biodiversity in Marine Benthic Communities. Oikos.

[19] Fabricius K.E., De’ath G., Noonan S., Uthicke S. (2014). Ecological Effects of Ocean Acidification and Habitat Complexity on Reef-Associated Macroinvertebrate Communities. Proc. R. Soc. B Biol. Sci..

[20] Young C.S., Gobler C.J. (2016). Ocean Acidification Accelerates the Growth of Two Bloom-Forming Macroalgae. PLoS ONE.

[21] Maibam C., Fink P., Romano G., Buia M.C., Butera E., Zupo V. (2015). Centropages Typicus (Crustacea, Copepoda) Reacts to Volatile Compounds Produced by Planktonic Algae. Mar. Ecol..

[22] Mutalipassi M., Mazzella V., Schott M., Fink P., Glaviano F., Porzio L., Lorenti M., Buia M.C., von Elert E., Zupo V. (2022). Ocean Acidification Affects Volatile Infochemicals Production and Perception in Fauna and Flora Associated with *Posidonia oceanica* (L.) Delile. Front. Mar. Sci..

[23] Price N.N., Hamilton S.L., Tootell J.S., Smith J.E. (2011). Species-Specific Consequences of Ocean Acidification for the Calcareous Tropical Green Algae Halimeda. Mar. Ecol. Prog. Ser..

[24] Gaubert J., Greff S., Thomas O.P., Payri C.E. (2019). Metabolomic Variability of Four Macroalgal Species of the Genus Lobophora Using Diverse Approaches. Phytochemistry.

[25] Kumar M., Kuzhiumparambil U., Pernice M., Jiang Z., Ralph P.J. (2016). Metabolomics: An Emerging Frontier of Systems Biology in Marine Macrophytes. Algal Res..

[26] Porzio L., Buia M.C., Hall-Spencer J.M. (2011). Effects of Ocean Acidification on Macroalgal Communities. J. Exp. Mar. Bio. Ecol..

[27] El-Sayed H.S., Elshobary M.E., Barakat K.M., Khairy H.M., El-Sheikh M.A., Czaja R., Allam B., Senousy H.H. (2022). Ocean Acidification Induced Changes in Ulva Fasciata Biochemistry May Improve Dicentrarchus Labrax Aquaculture via Enhanced Antimicrobial Activity. Aquaculture.

[28] Kumar A., Nonnis S., Castellano I., AbdElgawad H., Beemster G.T.S., Buia M.C., Maffioli E., Tedeschi G., Palumbo A. (2022). Molecular Response of Sargassum Vulgare to Acidification at Volcanic CO_2_ Vents: Insights from Proteomic and Metabolite Analyses Correspondence. Mol. Ecol..

[29] O’Leary J.K., Barry J.P., Gabrielson P.W., Rogers-Bennett L., Potts D.C., Palumbi S.R., Micheli F. (2017). Calcifying Algae Maintain Settlement Cues to Larval Abalone Following Algal Exposure to Extreme Ocean Acidification. Sci. Rep..

[30] Cornwall C.E., Hepburn C.D., Pritchard D., Currie K.I., Mcgraw C.M., Hunter K.A., Hurd C.L. (2012). Carbon-Use Strategies in Macroalgae: Differential Responses to Lowered Ph and Implications for Ocean Acidification. J. Phycol..

[31] Rautenberger R., Ferná ndez P.A., Strittmatter M., Heesch S., Cornwall C.E., Hurd C.L., Roleda M.Y., Michael Roleda C.Y., Norwegian B. (2015). Saturating Light and Not Increased Carbon Dioxide under Ocean Acidification Drives Photosynthesis and Growth in Ulva Rigida (Chlorophyta). Ecol. Evol..

[32] Gao K., Zhang Y., Häder D.P. (2018). Individual and Interactive Effects of Ocean Acidification, Global Warming, and UV Radiation on Phytoplankton. J. Appl. Phycol..

[33] Hurd C.L., Hepburn C.D., Currie K.I., Raven J.A., Hunter K.A. (2009). Testing the Effects of Ocean Acidification on Algal Metabolism: Considerations for Experimental Designs1. J. Phycol..

[34] Wang C., Jiao X., Zhang Y., Zhang L., Xu H. (2020). A Light-Limited Growth Model Considering the Nutrient Effect for Improved Understanding and Prevention of Macroalgae Bloom. Environ. Sci. Pollut. Res..

[35] He Y., Hu C., Wang Y., Cui D., Sun X., Li Y., Xu N. (2018). The Metabolic Survival Strategy of Marine Macroalga Ulva Prolifera under Temperature Stress. J. Appl. Phycol..

[36] Guan C., Zhao X., Qu T., Zhong Y., Hou C., Lin Z., Xu J., Tang X., Wang Y. (2022). Physiological Functional Traits Explain Morphological Variation of Ulva Prolifera during the Drifting of Green Tides. Ecol. Evol..

[37] Michalak I., Chojnacka K. (2009). Edible Macroalga Ulva Prolifera as Microelemental Feed Supplement for Livestock: The Fundamental Assumptions of the Production Method. World J. Microbiol. Biotechnol..

[38] Cai C., Liu X., Zhao H., Jiang T., Jia R., He P. (2021). Weakened Growth, Cell Division, and Energy Metabolism, but Enhanced Resistance, Signaling, and Anabolism: Responses of Ulva Prolifera to Copper Elucidated by Omics. J. Appl. Phycol..

[39] Guo F., Han M., Lin S., Ye H., Chen J., Zhu H., Lin W. (2021). Enteromorpha Prolifera Polysaccharide Prevents High- Fat Diet-Induced Obesity in Hamsters: A NMR-Based Metabolomic Evaluation. J. Food Sci..

[40] Lankadurai B.P., Nagato E.G., Simpson M.J. (2013). Environmental Metabolomics: An Emerging Approach to Study Organism Responses to Environmental Stressors. Environ. Rev..

[41] Bundy J.G., Davey M.P., Viant M.R. (2009). Environmental Metabolomics: A Critical Review and Future Perspectives. Metabolomics.

[42] Kikuchi J., Ito K., Date Y. (2018). Environmental Metabolomics with Data Science for Investigating Ecosystem Homeostasis. Prog. Nucl. Magn. Reson. Spectrosc..

[43] Schneider K.R. (2008). Heat Stress in the Intertidal: Comparing Survival and Growth of an Invasive and Native Mussel under a Variety of Thermal Conditions. Biol. Bull..

[44] Ghaderiardakani F., Langhans L., Kurbel V.B., Fenizia S., Wichard T. (2022). Metabolite Profiling Reveals Insights into the Species-Dependent Cold Stress Response of the Green Seaweed Holobiont Ulva (Chlorophyta). Environ. Exp. Bot..

[45] Liu L., Sanchez-Arcos C., Pohnert G., Wei D. (2021). Untargeted Metabolomics Unveil Changes in Autotrophic and Mixotrophic Galdieria Sulphuraria Exposed to High-Light Intensity. Int. J. Mol. Sci..

[46] Welker A.F., Moreira D.C., Campos É.G., Hermes-Lima M. (2013). Role of Redox Metabolism for Adaptation of Aquatic Animals to Drastic Changes in Oxygen Availability. Comp. Biochem. Physiol. Part A Mol. Integr. Physiol..

[47] Zhang M., Li L., Liu Y., Gao X. (2020). Effects of a Sudden Drop in Salinity on Scapharca Subcrenata Antioxidant Defenses and Metabolism Determined Using LC-MS Non-Targeted Metabolomics. Sci. Rep..

[48] Sogin E.M., Putnam H.M., Anderson P.E., Gates R.D. (2016). Metabolomic Signatures of Increases in Temperature and Ocean Acidification from the Reef-Building Coral, Pocillopora Damicornis. Metabolomics.

[49] Gaubert J., Rodolfo-Metalpa R., Greff S., Thomas O.P., Payri C.E. (2020). Impact of Ocean Acidification on the Metabolome of the Brown Macroalgae Lobophora Rosacea from New Caledonia. Algal Res..

[50] Tan Y.H., Lim P.E., Beardall J., Poong S.W., Phang S.M. (2019). A Metabolomic Approach to Investigate Effects of Ocean Acidification on a Polar Microalga *Chlorella* sp. Aquat. Toxicol..

[51] Trigg S.A., McElhany P., Maher M., Perez D., Busch D.S., Nichols K.M. (2019). Uncovering Mechanisms of Global Ocean Change Effects on the Dungeness Crab (Cancer Magister) through Metabolomics Analysis. Sci. Rep..

[52] Zunino S., Canu D.M., Zupo V., Solidoro C. (2019). Direct and Indirect Impacts of Marine Acidification on the Ecosystem Services Provided by Coralligenous Reefs and Seagrass Systems. Glob. Ecol. Conserv..

[53] Saha M., Berdalet E., Carotenuto Y., Fink P., Harder T., John U., Not F., Pohnert G., Potin P., Selander E. (2019). Using Chemical Language to Shape Future Marine Health. Front. Ecol. Environ..

[54] Peñuelas J., Sardans J. (2009). Ecological Metabolomics. Chem. Ecol..

[55] Nagler M., Nägele T., Gilli C., Fragner L., Korte A., Platzer A., Farlow A., Nordborg M., Weckwerth W. (2018). Eco-Metabolomics and Metabolic Modeling: Making the Leap from Model Systems in the Lab to Native Populations in the Field. Front. Plant Sci..

[56] Barakat K.M., El-Sayed H.S., Khairy H.M., El-Sheikh M.A., Al-Rashed S.A., Arif I.A., Elshobary M.E. (2021). Effects of Ocean Acidification on the Growth and Biochemical Composition of a Green Alga (Ulva Fasciata) and Its Associated Microbiota. Saudi J. Biol. Sci..

[57] Harrysson H., Konasani V.R., Toth G.B., Pavia H., Albers E., Undeland I. (2019). Strategies for Improving the Protein Yield in PH-Shift Processing of Ulva Lactuca Linnaeus: Effects of Ulvan Lyases, PH-Exposure Time, and Temperature. ACS Sustain. Chem. Eng..

[58] Gao K., Campbell D.A. (2014). Photophysiological Responses of Marine Diatoms to Elevated CO_2_ and Decreased PH: A Review. Funct. Plant Biol..

[59] Machado M., Machado S., Pimentel F.B., Freitas V., Alves R.C., Oliveira M.B.P.P. (2020). Amino Acid Profile and Protein Quality Assessment of Macroalgae Produced in an Integrated Multi-Trophic Aquaculture System. Foods.

[60] Kazir M., Abuhassira Y., Robin A., Nahor O., Luo J., Israel A., Golberg A., Livney Y.D. (2019). Extraction of Proteins from Two Marine Macroalgae, Ulva Sp. and Gracilaria Sp., for Food Application, and Evaluating Digestibility, Amino Acid Composition and Antioxidant Properties of the Protein Concentrates. Food Hydrocoll..

[61] Gaufichon L., Rothstein S.J., Suzuki A. (2016). Asparagine Metabolic Pathways in Arabidopsis. Plant Cell Physiol..

[62] Weinberger F., Pohnert G., Berndt M.L., Bouarab K., Kloareg B., Potin P. (2005). Apoplastic Oxidation of L-Asparagine Is Involved in the Control of the Green Algal Endophyte Acrochaete Operculata Correa & Nielsen by the Red Seaweed Chondrus Crispus Stackhouse. J. Exp. Bot..

[63] Robinson S.A., Slade A.P., Fox G.G., Phillips R., Ratcliffe R.G., Stewart G.R. (1991). The Role of Glutamate Dehydrogenase in Plant Nitrogen Metabolism. Plant Physiol..

[64] Bascomb N.F., Schmidt R.R. (1987). Purification and Partial Kinetic and Physical Characterization of Two Chloroplast-Localized NADP-Specific Glutamate Dehydrogenase Isoenzymes and Their Preferential Accumulation in Chlorella Sorokiniana Cells Cultured at Low or High Ammonium Levels.

[65] Kakinuma M., Coury D.A., Kuno Y., Itoh S., Kozawa Y., Inagaki E., Yoshiura Y., Amano H. (2006). Physiological and Biochemical Responses to Thermal and Salinity Stresses in a Sterile Mutant of Ulva Pertusa (Ulvales, Chlorophyta). Mar. Biol..

[66] Muñoz-Blanco J., Moyano E., Cárdenas J. (1989). Glutamate Dehydrogenase Isozymes of Chlamydomonas Reinhardtii. FEMS Microbiol. Lett..

[67] Inokuchi R., Itagaki T., Wiskich J.T., Nakayama K., Okada M. (1997). An NADP-Glutamate Dehydrogenase from the Green Alga Bryopsis Maxima. Purification and Properties. Plant Cell Physiol..

[68] Sato M., Sato Y., Tsuchiya Y. (1984). Glutamate Dehydrogenase of Porphyra Yezoensis. Hydrobiologia.

[69] Kumar A., Bera S. (2020). Revisiting Nitrogen Utilization in Algae: A Review on the Process of Regulation and Assimilation. Bioresour. Technol. Rep..

[70] Liu X., Huan Z., Zhang Q., Zhong M., Chen W., Aslam M., Du H. (2019). Glutamine Synthetase (GS): A Key Enzyme for Nitrogen Assimilation in The Macroalga Gracilariopsis Lemaneiformis (Rhodophyta). J. Phycol..

[71] Chellamuthu V.R., Ermilova E., Lapina T., Lüddecke J., Minaeva E., Herrmann C., Hartmann M.D., Forchhammer K. (2014). A Widespread Glutamine-Sensing Mechanism in the Plant Kingdom. Cell.

[72] Kirst G.O. (1996). Osmotic Adjustment in Phytoplankton and MacroAlgae. Biological and Environmental Chemistry of DMSP and Related Sulfonium Compounds.

[73] Street T.O., Bolen D.W., Rose G.D. (2006). A Molecular Mechanism for Osmolyte-Induced Protein Stability. Proc. Natl. Acad. Sci..

[74] Kumar A., Buia M.C., Palumbo A., Mohany M., Wadaan M.A.M., Hozzein W.N., Beemster G.T.S., AbdElgawad H. (2020). Ocean Acidification Affects Biological Activities of Seaweeds: A Case Study of Sargassum Vulgare from Ischia Volcanic CO_2_ Vents. Environ. Pollut..

[75] Ruan Y.L. (2012). Signaling Role of Sucrose Metabolism in Development. Mol. Plant.

[76] Koch K. (2004). Sucrose Metabolism: Regulatory Mechanisms and Pivotal Roles in Sugar Sensing and Plant Development. Curr. Opin. Plant Biol..

[77] Wippel K., Wittek A., Hedrich R., Sauer N. (2010). Inverse Ph Regulation of Plant and Fungal Sucrose Transporters: A Mechanism to Regulate Competition for Sucrose at the Host/Pathogen Interface?. PLoS ONE.

[78] Kolman M.A., Nishi C.N., Perez-Cenci M., Salerno G.L. (2015). Sucrose in Cyanobacteria: From a Salt-Response Molecule to Play a Key Role in Nitrogen Fixation. Life.

[79] Pogoreutz C., Rädecker N., Cárdenas A., Gärdes A., Voolstra C.R., Wild C. (2017). Sugar Enrichment Provides Evidence for a Role of Nitrogen Fixation in Coral Bleaching. Glob. Chang. Biol..

[80] Fu W., Gudmundsson S., Wichuk K., Palsson S., Palsson B.O., Salehi-Ashtiani K., Brynjólfsson S. (2019). Sugar-Stimulated CO_2_ Sequestration by the Green Microalga Chlorella Vulgaris. Sci. Total Environ..

[81] Mo’o F.R.C., Wilar G., Devkota H.P., Wathoni N. (2020). Ulvan, a Polysaccharide from Macroalga Ulva Sp.: A Review of Chemistry, Biological Activities and Potential for Food and Biomedical Applications. Appl. Sci..

[82] Kidgell J.T., Magnusson M., de Nys R., Glasson C.R.K. (2019). Ulvan: A Systematic Review of Extraction, Composition and Function. Algal Res..

[83] Lahaye M., Jegou D. (1993). Chemical and Physical-Chemical Characteristics of Dietary Fibres from *Ulva lactuca* (L.) Thuret and *Enteromorpha compressa* (L.) Grev. J. Appl. Phycol..

[84] Silva T.H., Alves A., Popa E.G., Reys L.L., Gomes M.E., Sousa R.A., Silva S.S., Mano J.F., Reis R.L. (2012). Marine Algae Sulfated Polysaccharides for Tissue Engineering and Drug Delivery Approaches. Biomatter.

[85] Angelova M.I., Bitbol A.F., Seigneuret M., Staneva G., Kodama A., Sakuma Y., Kawakatsu T., Imai M., Puff N. (2018). PH Sensing by Lipids in Membranes: The Fundamentals of PH-Driven Migration, Polarization and Deformations of Lipid Bilayer Assemblies. Biochim. Biophys. Acta-Biomembr..

[86] Akimov S.A., Polynkin M.A., Jiménez-Munguía I., Pavlov K.V., Batishchev O.V. (2018). Phosphatidylcholine Membrane Fusion Is PH-Dependent. Int. J. Mol. Sci..

[87] Casey J.R., Grinstein S., Orlowski J. (2010). Sensors and Regulators of Intracellular PH. Nat. Rev. Mol. Cell Biol..

[88] Gross W. (2000). Ecophysiology of Algae Living in Highly Acidic Environments. Hydrobiologia.

[89] Catalanotti C., Yang W., Posewitz M.C., Grossman A.R. (2013). Fermentation Metabolism and Its Evolution in Algae. Front. Plant Sci..

[90] Putnam R.W. (2012). Intracellular PH Regulation. Cell Physiology Source Book.

[91] Hirooka S., Hirose Y., Kanesaki Y., Higuchi S., Fujiwara T., Onuma R., Era A., Ohbayashi R., Uzuka A., Nozaki H. (2017). Acidophilic Green Algal Genome Provides Insights into Adaptation to an Acidic Environment. Proc. Natl. Acad. Sci. USA.

[92] Messerli M.A., Amaral-Zettler L.A., Zettler E., Jung S.K., Smith P.J.S., Sogin M.L. (2005). Life at Acidic PH Imposes an Increased Energetic Cost for a Eukaryotic Acidophile. J. Exp. Biol..

[93] Yoshida M., Ioki M., Matsuura H., Hashimoto A., Kaya K., Nakajima N., Watanabe M.M. (2020). Diverse Steroidogenic Pathways in the Marine Alga Aurantiochytrium. J. Appl. Phycol..

[94] Benveniste P. (2004). Biosynthesis and Accumulation of Sterols. Annu. Rev. Plant Biol..

[95] Hannich J.T., Umebayashi K., Riezman H. (2011). Distribution and Functions of Sterols and Sphingolipids. Cold Spring Harb. Perspect. Biol..

[96] Ohyama K., Suzuki M., Kikuchi J., Saito K., Muranaka T. (2009). Dual Biosynthetic Pathways to Phytosterol via Cycloartenol and Lanosterol in Arabidopsis. Proc. Natl. Acad. Sci. USA.

[97] Gu K., Liu Y., Jiang T., Cai C., Zhao H., Liu X., He P. (2022). Effect of a Short-Term Light Stress on Resistance, Signaling, Metabolism, and Cell Division of Ulva Prolifera Revealed by Omics.

[98] Kumari P., Kumar M., Reddy C.R.K., Jha B. (2013). Algal Lipids, Fatty Acids and Sterols. Functional Ingredients from Algae for Foods and Nutraceuticals.

[99] Stefels J. (2000). Physiological Aspects of the Production and Conversion of DMSP in Marine Algae and Higher Plants. J. Sea Res..

[100] Kirst G.O., Thiel C., Wolff H., Nothnagel J., Wanzek M., Ulmke R. (1991). Dimethylsulfoniopropionate (DMSP) in Icealgae and Its Possible Biological Role. Mar. Chem..

[101] Kessler R.W., Weiss A., Kuegler S., Hermes C., Wichard T. (2018). Macroalgal-Bacterial Interactions: Role of Dimethylsulfoniopropionate in Microbial Gardening by Ulva (Chlorophyta). Mol. Ecol..

[102] Steinke M., Malin G., Liss P.S. (2002). Trophic Interactions in the Sea: An Ecological Role for Climate Relevant Volatiles?. J. Phycol..

[103] Van Alstyne K.L., Puglisi M.P. (2007). DMSP in Marine Macroalgae and Macroinvertebrates: Distribution, Function, and Ecological Impacts. Aquat. Sci..

[104] Saint-Macary A.D., Barr N., Armstrong E., Safi K., Marriner A., Gall M., McComb K., Dillingham P.W., Law C.S. (2021). The Influence of Ocean Acidification and Warming on Dmsp & Dms in New Zealand Coastal Water. Atmosphere.

[105] Hopkins F.E., Nightingale P.D., Stephens J.A., Moore C.M., Richier S., Cripps G.L., Archer S.D. (2018). Dimethylsulfide (DMS) Production in Polar Oceans May Be Resilient to Ocean Acidification. Biogeosciences Discuss.

[106] Bénard R., Levasseur M., Scarratt M., Michaud S., Starr M., Mucci A., Ferreyra G., Gosselin M., Tremblay J.É., Lizotte M. (2019). Contrasting Effects of Acidification and Warming on Dimethylsulfide Concentrations during a Temperate Estuarine Fall Bloom Mesocosm Experiment. Biogeosciences.

[107] Avgoustidi V., Nightingale P.D., Joint I., Steinke M., Turner S.M., Hopkins F.E., Liss P.S. (2012). Decreased Marine Dimethyl Sulfide Production under Elevated CO_2_ Levels in Mesocosm and in Vitro Studies. Environ. Chem..

[108] Archer S.D., Kimmance S.A., Stephens J.A., Hopkins F.E., Bellerby R.G.J., Schulz K.G., Piontek J., Engel A. (2013). Contrasting Responses of DMS and DMSP to Ocean Acidification in Arctic Waters. Biogeosciences.

[109] Zhang H., Zou J., Yan X., Chen J., Cao X., Wu J., Liu Y., Wang T. (2021). Marine-Derived Macrolides 1990–2020: An Overview of Chemical and Biological Diversity. Mar. Drugs.

[110] Chen J., Lv S., Liu J., Yu Y., Wang H., Zhang H. (2021). An Overview of Bioactive 1,3-Oxazole-Containing Alkaloids from Marine Organisms. Pharmaceuticals.

[111] Pawlik J.R., Kernan M.R., Molinski T.F., Harper M.K., Faulkner D.J. (1988). Defensive Chemicals of the Spanisch Dancer Nudibranch Hexabranchus Sanguineus and Its Egg Ribbons: Macrolides Derived from a Sponge Diet. J. Exp. Mar. Bio. Ecol..

[112] Scheuer P.J. (1990). Some Marine Ecological Phenomena: Chemical Basis and Biomedical Potential. Science.

[113] Vítová M., Goecke F., Sigler K., Řezanka T. (2016). Lipidomic Analysis of the Extremophilic Red Alga Galdieria Sulphuraria in Response to Changes in PH. Algal Res..

[114] Jin P., Hutchins D.A., Gao K. (2020). The Impacts of Ocean Acidification on Marine Food Quality and Its Potential Food Chain Consequences. Front. Mar. Sci..

[115] Jin P., Liang Z., Lu H., Pan J., Li P., Huang Q., Guo Y., Zhong J., Li F., Wan J. (2021). Lipid Remodeling Reveals the Adaptations of a Marine Diatom to Ocean Acidification. Front. Microbiol..

[116] Boudière L., Michaud M., Petroutsos D., Rébeillé F., Falconet D., Bastien O., Roy S., Finazzi G., Rolland N., Jouhet J. (2014). Glycerolipids in Photosynthesis: Composition, Synthesis and Trafficking. Biochim. Biophys. Acta-Bioenerg..

[117] Mikami K. (2014). Structural Divergence and Loss of Phosphoinositide-Specific Phospholipase C Signaling Components during the Evolution of the Green Plant Lineage: Implications from Structural Characteristics of Algal Components. Front. Plant Sci..

[118] Tatsuzawa H., Takizawa E., Wada M., Yamamoto Y. (1996). Fatty Acid and Lipid Composition of the Acidophilic Green Alga *Chlamydomonas* sp. J. Phycol..

[119] Bermúdez J.R., Riebesell U., Larsen A., Winder M. (2016). Ocean Acidification Reduces Transfer of Essential Biomolecules in a Natural Plankton Community. Sci. Rep..

[120] Rossoll D., Bermúdez R., Hauss H., Schulz K.G., Riebesell U., Sommer U., Winder M. (2012). Ocean Acidification-Induced Food Quality Deterioration Constrains Trophic Transfer. PLoS ONE.

[121] Kumar A., AbdElgawad H., Castellano I., Selim S., Beemster G.T.S., Asard H., Buia M.C., Palumbo A. (2018). Effects of Ocean Acidification on the Levels of Primary and Secondary Metabolites in the Brown Macroalga Sargassum Vulgare at Different Time Scales. Sci. Total Environ..

[122] Sato N., Tsuzuki M., Kawaguchi A. (2003). Glycerolipid Synthesis in Chlorella Kessleri 11 h-II. Effect of the CO_2_ Concentration during Growth. Biochim. Biophys. Acta-Mol. Cell Biol. Lipids.

[123] Zupo V., Buia M.C., Mazzella L. (1997). A Production Model for Posidonia Oceanica Based on Temperature. Estuar. Coast. Shelf Sci..

[124] Cui J., Monotilla A.P., Zhu W., Takano Y., Shimada S., Ichihara K., Matsui T., He P., Hiraoka M. (2019). Taxonomic Reassessment of Ulva Prolifera (Ulvophyceae, Chlorophyta) Based on Specimens from the Type Locality and Yellow Sea Green Tides. Phycologia.

[125] Guillard R.R.L. (1975). Culture of Phytoplankton for Feeding Marine Invertebrates. Culture of Marine Invertebrate Animals.

[126] Gattuso J.-P., Lee K., Rost B., Schulz K. (2010). Approaches and Tools to Manipulate the Carbonate Chemistry.

[127] Hwang T.L., Shaka A.J. (1995). Water Suppression That Works. Excitation Sculpting Using Arbitrary Wave-Forms and Pulsed-Field Gradients. J. Magn. Reson.-Ser. A.

[128] Griesinger C., Otting G., Wüthrich K., Ernst R.R. (1988). Clean Tocsy for 1H Spin System Identification in Macromolecules. J. Am. Chem. Soc..

[129] Bax A., Davis D.G. (1985). MLEV-17-Based Two-Dimensional Homonuclear Magnetization Transfer Spectroscopy. J. Magn. Reson..

[130] Palmer A.G., Cavanagh J., Wright P.E., Rance M. (1991). Sensitivity Improvement in Proton-Detected Two-Dimensional Heteronuclear Correlation NMR Spectroscopy. J. Magn. Reson..

[131] Kay L.E., Keifer P., Saarinen T. (1992). Pure Absorption Gradient Enhanced Heteronuclear Single Quantum Correlation Spectroscopy with Improved Sensitivity. J. Am. Chem. Soc..

[132] Schleucher J., Schwendinger M., Sattler M., Schmidt P., Schedletzky O., Glaser S.J., Sørensen O.W., Griesinger C. (1994). A General Enhancement Scheme in Heteronuclear Multidimensional NMR Employing Pulsed Field Gradients. J. Biomol. NMR.

[133] Fan T.W.M. (1996). Metabolite Profiling by One- and Two-Dimensional NMR Analysis of Complex Mixtures. Prog. Nucl. Magn. Reson. Spectrosc..

[134] Wishart D.S., Guo A.C., Oler E., Wang F., Anjum A., Peters H., Dizon R., Sayeeda Z., Tian S., Lee B.L. (2022). HMDB 5.0: The Human Metabolome Database for 2022. Nucleic Acids Res..

[135] Picart-Armada S., Fernández-Albert F., Vinaixa M., Rodríguez M.A., Aivio S., Stracker T.H., Yanes O., Perera-Lluna A. (2017). Null Diffusion-Based Enrichment for Metabolomics Data. PLoS ONE.

[136] Picart-Armada S., Fernández-Albert F., Vinaixa M., Yanes O., Perera-Lluna A. (2018). FELLA: An R Package to Enrich Metabolomics Data. BMC Bioinform..

[137] Kessner D., Chambers M., Burke R., Agus D., Mallick P. (2008). ProteoWizard: Open Source Software for Rapid Proteomics Tools Development. Bioinformatics.

[138] Benton H.P., Want E.J., Ebbels T.M.D. (2010). Correction of Mass Calibration Gaps in Liquid Chromatography-Mass Spectrometry Metabolomics Data. Bioinformatics.

[139] Smith C.A., Want E.J., O’Maille G., Abagyan R., Siuzdak G. (2006). XCMS: Processing Mass Spectrometry Data for Metabolite Profiling Using Nonlinear Peak Alignment, Matching, and Identification. Anal. Chem..

[140] Tautenhahn R., Bottcher C., Neumann S. (2008). Highly Sensitive Feature Detection for High Resolution LC/MS. BMC Bioinform..

[141] Kuhl C., Tautenhahn R., Böttcher C., Larson T.R., Neumann S. (2012). CAMERA: An Integrated Strategy for Compound Spectra Extraction and Annotation of Liquid Chromatography/Mass Spectrometry Data Sets. Anal. Chem..

[142] Chong J., Yamamoto M., Xia J. (2019). MetaboAnalystR 2.0: From Raw Spectra to Biological Insights. Metabolites.

[143] Rajan K., Zielesny A., Steinbeck C. (2021). STOUT: SMILES to IUPAC Names Using Neural Machine Translation. J. Cheminform..

[144] Cutignano A., Luongo E., Nuzzo G., Pagano D., Manzo E., Sardo A., Fontana A. (2016). Profiling of Complex Lipids in Marine Microalgae by UHPLC/Tandem Mass Spectrometry. Algal Res..

[145] Matyash V., Liebisch G., Kurzchalia T.V., Shevchenko A., Schwudke D. (2008). Lipid Extraction by Methyl-Terf-Butyl Ether for High-Throughput Lipidomics. J. Lipid Res..

[146] Cutignano A., Mamone G., Boscaino F., Ceriotti A., Maccaferri M., Picariello G. (2021). Monitoring Changes of Lipid Composition in Durum Wheat during Grain Development. J. Cereal Sci..

[147] Suleria H.A.R., Osborne S., Masci P., Gobe G. (2015). Marine-Based Nutraceuticals: An Innovative Trend in the Food and Supplement Industries. Mar. Drugs.

