# Supplementary material for: Responses of the Macroalga Ulva prolifera Müller to Ocean Acidification Revealed by Complementary NMR- and MS-Based Omics Approaches
Source: Mar Drugs. 2022 Nov 26;20(12):743. doi: 10.3390/md20120743 (PMC9783899; doi:10.3390/md20120743)
Supplement: Supplementary file 1 [file marinedrugs-20-00743-s001.zip › marinedrugs-1951729-supplementary_rev.pdf]

SUPPLEMENTARY MATERIAL

Responses of the macroalga *Ulva prolifera* Müller to ocean acidification revealed by complementary NMR- and MS-based omics approaches

Carlos Sanchez-Arcos [1,†], Debora Paris [2,†], Valerio Mazzella [3], Mirko Mutalipassi [4], Maria Costantini [5], Maria Cristina Buia [6], Eric von Elert [1], Adele Cutignano [2,5,*], Valerio Zupo [6,*]

1 Institute for Zoology, Cologne Biocenter University of Cologne, 50674 Köln, Germany
2 Consiglio Nazionale delle Ricerche (CNR), Istituto di Chimica Biomolecolare (ICB), 80078 Pozzuoli (NA), Italy
3 Stazione Zoologica Anton Dohrn, Department of Marine Integrated Ecology, Ischia Marine Center, 80077 Ischia (NA), Italy
4 Stazione Zoologica Anton Dohrn, Department of Integrative Marine Ecology, Stazione Zoologica Anton Dohrn, Calabria Marine Centre, 87071 Amendolara (CS), Italy
5 Stazione Zoologica Anton Dohrn, Department of Ecosustainable Marine Biotechnology, 80121 Napoli, Italy
6 Stazione Zoologica Anton Dohrn, Department of Ecosustainable Marine Biotechnology, 80077 Ischia (NA), Italy.

† These authors equally contributed.
* Correspondence: acutignano@icb.cnr.it; Tel.: +39 0818675313 (AC) ; vzupo@szn.it; Tel: +39 081 5833503 (VZ)

Table S1. Parameters of the logistic growth evaluated for *Ulva prolifera* cultivated at two pH regimes.

Table S2. *Ulva prolifera* enrichment analysis.

Figure S1. MS^2 spectra of *m/z* 452.8875, $[M+Cl]^-$ adduct of compound 145.

Figure S2. MS^2 spectra of *m/z* 695.3517, $[M-2H+Na]^-$ adduct of compound 9.

Figure S3. MS^2 spectra of *m/z* 475.3714, $[M+Cl]^-$ adduct of compound 66.

Figure S4. MS^2 spectra of *m/z* 617.3595 (unknown compound 103).

Figure S5. MS^2 spectra of *m/z* 528.3198, $[M-H]^-$ ion of compound 151

Figure S6. Lipid yield after *Ulva prolifera* extraction by MTBE/MeOH. Values are reported as a percentage (%) of dry weight ± SD of four replicates in the two growth conditions.

Figure S7. Class profile lipidome of *Ulva prolifera* grown in normal (pH 8.1, replicates c, s1-s3) *vs* acidic (pH 7.7, replicates s4-s7) conditions. The data shown were not normalized.

Figure S8. Phosphatidylcholine (PC) composition and semi-quantitation in *Ulva prolifera* grown at pH 8.1 and pH 7.7. Values are reported as ng/mg DW ± SD (n= 4).

Figure S9. Phosphatidylglycerol (PG) composition and semi-quantitation in *Ulva prolifera* grown at pH 8.1 and pH 7.7. Values are reported as ng/mg DW ± SD (n= 4).

Figure S10. Phosphatidylethanolamine (PE) composition and semi-quantitation in *Ulva prolifera* grown at pH 8.1 and pH 7.7. Values are reported as ng/mg DW ± SD (n= 4).

Figure S11. Monogalactosyldiacylglycerol (MGDG) composition and semi-quantitation in *Ulva prolifera* grown at pH 8.1 and pH 7.7. Values are reported as ng/mg DW ± SD (n= 4).

Figure S12. Digalactosyldiacylglycerol (DGDG) composition and semi-quantitation in *Ulva prolifera* grown at pH 8.1 and pH 7.7. Values are reported as ng/mg DW ± SD (n= 4).

Figure S13. Sulfoquinovosyldiacylglycerol (SQDG) composition and semi-quantitation in *Ulva prolifera* grown at pH 8.1 and pH 7.7. Values are reported as ng/mg DW ± SD (n= 4).

Figure S14. Phosphatidylinositol (PI) composition and semi-quantitation in *Ulva prolifera* grown at pH 8.1 and pH 7.7. Values are reported as ng/mg DW ± SD (n= 4).

Methods S1. Growth curves' logistic equation.

Methods S2. NMR spectra acquisition and parameters.

Table S1. Parameters of the logistic growth evaluated for *Ulva prolifera* cultivated at two pH regimes.

	Control 8.1	Acidified 7.7
Logistic growth		
Best-fit values		
Y_M	32,10	61,37
Y_0	3,736	5,472
k	0,2326	0,08518
X_{int}	4,300	11,74
95% CI (profile likelihood)		
Y_M	29,59 to 34,84	52,44 to 83,04
Y_0	0,8157 to 7,702	2,519 to 9,371
k	0,1370 to 0,4713	0,05619 to 0,1214
X_{int}	2,122 to 7,299	8,238 to 17,80
Goodness of Fit		
Degrees of Freedom	49	46
R squared	0,7090	0,8278
Sum of Squares	2210	3130
Sy.x	6,716	8,249
Number of points		
# of X values	52	52
# Y values analyzed	52	49

Table S2. *Ulva prolifera* enrichment analysis.

KEGG id	Entry type	KEGG name	p score
cre00220	pathway	Arginine biosynthesis - Chlamydomonas reinhar...	1,1984E-02
cre00250	pathway	Alanine, aspartate and glutamate metabolism -...	1,0000E-06
cre00330	pathway	Arginine and proline metabolism - Chlamydomon...	1,0000E-06
cre00500	pathway	Starch and sucrose metabolism - Chlamydomonas...	1,5328E-03
cre00670	pathway	One carbon pool by folate - Chlamydomonas rei...	3,9828E-02
cre00920	pathway	Sulfur metabolism - Chlamydomonas reinhardtii	1,0000E-06
cre02010	pathway	ABC transporters - Chlamydomonas reinhardtii	3,6294E-02
M00029	module	Urea cycle	7,1211E-04
M00133	module	Polyamine biosynthesis, arginine => agmatine ...	7,0291E-03
M00134	module	Polyamine biosynthesis, arginine => ornithine...	1,0000E-06
M00555	module	Betaine biosynthesis, choline => betaine	1,0000E-06
M00596	module	Dissimilatory sulfate reduction, sulfate => H...	3,1037E-02
M00616	module	Sulfate-sulfur assimilation	3,9000E-06
M00844	module	Arginine biosynthesis, ornithine => arginine	1,1328E-02
M00855	module	Glycogen degradation, glycogen => glucose-6P	3,0181E-02
1.13.11.53	enzyme	acireductone dioxygenase (Ni2+-requiring)	1,3711E-02
1.14.11.2	enzyme	procollagen-proline 4-dioxygenase	1,8466E-02
1.2.1.8	enzyme	betaine-aldehyde dehydrogenase	7,0627E-03
1.4.1.14	enzyme	glutamate synthase (NADH)	1,0000E-06
1.4.1.3	enzyme	glutamate dehydrogenase [NAD(P)+]	2,1161E-02
1.4.7.1	enzyme	glutamate synthase (ferredoxin)	1,0000E-06
1.5.1.54	enzyme	methylenetetrahydrofolate reductase (NADH)	4,7939E-03
1.8.3.1	enzyme	sulfite oxidase	3,1849E-03
1.8.3.4	enzyme	methanethiol oxidase	7,0033E-03
1.8.4.9	enzyme	adenylyl-sulfate reductase (glutathione)	4,5403E-02
2.1.3.3	enzyme	ornithine carbamoyltransferase	2,4967E-02
2.4.1.82	enzyme	galactinol---sucrose galactosyltransferase	1,0000E-06
2.6.1.13	enzyme	ornithine aminotransferase	1,7316E-02
2.6.1.16	enzyme	glutamine---fructose-6-phosphate transaminase...	1,1000E-06
2.6.1.2	enzyme	alanine transaminase	3,2324E-03
2.6.1.4	enzyme	glycine transaminase	7,3257E-03
2.6.1.85	enzyme	aminodeoxychorismate synthase	5,9257E-03
2.7.1.78	enzyme	polynucleotide 5'-hydroxyl-kinase	2,5742E-02
2.7.7.5	enzyme	sulfate adenylyltransferase (ADP)	1,2805E-02
3.1.4.46	enzyme	glycerophosphodiester phosphodiesterase	2,3061E-02
3.2.1.20	enzyme	alpha-glucosidase	1,9378E-02
3.2.1.26	enzyme	beta-fructofuranosidase	1,6198E-02
3.5.1.4	enzyme	amidase	9,0800E-05

4.1.1.17	enzyme	ornithine decarboxylase	2,1461E-02
4.1.3.27	enzyme	anthranilate synthase	2,0641E-02
4.3.2.1	enzyme	argininosuccinate lyase	1,1012E-02
4.3.3.6	enzyme	pyridoxal 5'-phosphate synthase (glutamine hy...	3,2420E+09
6.1.1.22	enzyme	asparagine---tRNA ligase	1,0000E-06
6.3.1.2	enzyme	glutamine synthetase	1,0000E-06
6.3.4.2	enzyme	CTP synthase (glutamine hydrolyzing)	1,0000E-06
6.3.4.3	enzyme	formate---tetrahydrofolate ligase	3,9159E-02
6.3.5.2	enzyme	GMP synthase (glutamine-hydrolyzing)	1,2033E+09
6.3.5.4	enzyme	asparagine synthase (glutamine-hydrolyzing)	1,0000E-06
6.3.5.5	enzyme	carbamoyl-phosphate synthase (glutamine-hydro...	6,7422E+09
6.3.5.6	enzyme	asparaginyl-tRNA synthase (glutamine-hydrolys...	1,3147E-02
6.3.5.7	enzyme	glutaminyl-tRNA synthase (glutamine-hydrolysi...	4,0661E-02
7.3.2.3	enzyme	ABC-type sulfate transporter	3,9009E+08
7.6.2.2	enzyme	ABC-type xenobiotic transporter	3,6294E-02
7.6.2.3	enzyme	ABC-type glutathione-S-conjugate transporter	3,6294E-02
R00021	reaction	L-glutamate:ferredoxin oxidoreductase (transa...	1,0000E-06
R00093	reaction	L-glutamate:NAD+ oxidoreductase (transaminati...	1,0000E-06
R00114	reaction	L-glutamate:NADP+ oxidoreductase (transaminat...	1,0000E-06
R00243	reaction	L-glutamate:NAD+ oxidoreductase (deaminating)	1,6323E-04
R00248	reaction	L-glutamate:NADP+ oxidoreductase (deaminating...	1,6077E-03
R00253	reaction	L-Glutamate:ammonia ligase (ADP-forming)	1,0000E-06
R00256	reaction	L-glutamine amidohydrolase	1,0000E-06
R00257	reaction	Deamido-NAD+:L-glutamine amido-ligase (AMP-fo...	2,6524E-04
R00258	reaction	L-Alanine:2-oxoglutarate aminotransferase	1,1628E-04
R00260	reaction	glutamate racemase	4,9521E-02
R00261	reaction	L-glutamate 1-carboxy-lyase (4-aminobutanoate...	8,0998E-03
R00355	reaction	L-Aspartate:2-oxoglutarate aminotransferase	2,1359E+09
R00372	reaction	Glycine:2-oxoglutarate aminotransferase	5,4315E-03
R00397	reaction	L-aspartate 4-carboxy-lyase (L-alanine-formin...	2,5239E-02
R00400	reaction	L-alanine:oxaloacetate aminotransferase	4,8741E-02
R00483	reaction	L-aspartate:ammonia ligase (AMP-forming)	1,0000E-06
R00485	reaction	L-asparagine amidohydrolase	1,0000E-06
R00551	reaction	L-Arginine amidinohydrolase	1,0000E-06
R00552	reaction	L-Arginine iminohydrolase	1,0000E-06
R00557	reaction	L-arginine,NADPH:oxygen oxidoreductase (nitri...	1,5222E+08
R00558	reaction	L-arginine,NADPH:oxygen oxidoreductase (N-(om...	1,0000E-06
R00559	reaction	L-Arginine:oxygen 2-oxidoreductase (decarboxy...	2,6864E-02
R00562	reaction	N2-(D-1-carboxyethyl)-L-arginine:NAD+ oxidore...	9,8816E-03
R00563	reaction	N2-(D-1,3-dicarboxypropyl)-L-arginine:NADP+ o...	9,1854E-03

R00565	reaction	L-Arginine:glycine amidinotransferase	1,0821E-03
R00566	reaction	L-arginine carboxy-lyase (agmatine-forming)	1,0633E-04
R00567	reaction	arginine racemase	8,6876E+08
R00571	reaction	UTP:ammonia ligase (ADP-forming)	4,1311E-02
R00573	reaction	UTP:L-glutamine amido-ligase (ADP-forming)	1,0000E-06
R00575	reaction	HCO3-:L-glutamine amido-ligase (ADP-forming, ...	1,0000E-06
R00576	reaction	L-Glutamine:pyruvate aminotransferase	6,1348E+08
R00578	reaction	L-aspartate:L-glutamine amido-ligase (AMP-for...	1,0000E-06
R00579	reaction	glutamine racemase	4,6745E-02
R00760	reaction	ATP:D-fructose 6-phosphotransferase	7,7749E-03
R00768	reaction	L-glutamine:D-fructose-6-phosphate isomerase ...	1,0000E-06
R00801	reaction	sucrose glucohydrolase	1,0000E-06
R00803	reaction	sucrose:phosphate alpha-D-glucosyltransferase	1,0000E-06
R00805	reaction	sucrose-6F-phosphate phosphohydrolase	1,0997E+08
R00806	reaction	UDP-glucose:D-fructose 2-alpha-D-glucosyltran...	1,0000E-06
R00807	reaction	Sucrose:(acceptor) 3-oxidoreductase	4,7645E-03
R00811	reaction	protein-Npi-phospho-L-histidine:sugar phospho...	7,1836E+08
R00832	reaction	succinyl-CoA:L-arginine N2-succinyltransferas...	2,8095E-02
R00867	reaction	ATP:D-fructose 6-phosphotransferase	1,0664E-02
R00878	reaction	alpha-D-Glucose aldose-ketose-isomerase	3,6682E-02
R00908	reaction	beta-alanine:2-oxoglutarate aminotransferase	4,7904E-02
R00912	reaction	L-arginine:beta-alanine ligase (ADP-forming)	2,9820E-02
R00943	reaction	Formate:tetrahydrofolate ligase (ADP-forming)	3,9511E-02
R00945	reaction	5,10-Methylenetetrahydrofolate:glycine hydrox...	3,0056E-02
R00946	reaction	5-methyltetrahydrofolate:L-homocysteine S-met...	1,3378E-03
R00986	reaction	Chorismate pyruvate-lyase (amino-accepting)	1,0000E-06
R01021	reaction	ATP:choline phosphotransferase	1,0000E-06
R01022	reaction	choline:oxygen 1-oxidoreductase	1,0000E-06
R01023	reaction	Acetyl-CoA:choline O-acetyltransferase	3,0328E-04
R01025	reaction	choline:acceptor 1-oxidoreductase	1,0000E-06
R01026	reaction	Acetylcholine aectylhydrolase	2,6975E-04
R01030	reaction	sn-Glycero-3-phosphocholine glycerophosphohyd...	1,0000E-06
R01072	reaction	5-phosphoribosylamine:diphosphate phospho-alp...	2,2550E-03
R01086	reaction	2-(Nomega-L-arginino)succinate arginine-lyase...	2,5353E-02
R01103	reaction	Raffinose galactohydrolase	1,0000E-06
R01217	reaction	5-methyltetrahydrofolate:ferredoxin oxidoredu...	1,4290E-03
R01224	reaction	5-methyltetrahydrofolate:NADP+ oxidoreductase	1,4306E-03
R01231	reaction	Xanthosine-5'-phosphate:L-glutamine amido-lig...	2,5322E+08
R01267	reaction	L-asparagine hydro-lyase (3-cyanoalanine-form...	1,0000E-06
R01310	reaction	phosphatidylcholine phosphatidohydrolase	1,0000E-06

R01346	reaction	L-asparagine:2-oxo-acid aminotransferase	1,0000E-06
R01566	reaction	Creatine amidinohydrolase	1,4212E-04
R01648	reaction	4-aminobutanoate:2-oxoglutarate aminotransfer...	3,0039E-02
R01716	reaction	chorismate:L-glutamine aminotransferase	3,2423E-03
R01823	reaction	Sucrose:1,4-alpha-D-glucan 4-alpha-D-glucosyl...	1,0000E-06
R01881	reaction	ATP:creatine N-phosphotransferase	1,0000E-06
R01883	reaction	S-Adenosyl-L-methionine:guanidinoacetate N-me...	5,5450E-03
R01884	reaction	Creatinine amidohydrolase	1,0000E-06
R01989	reaction	L-Arginine:4-aminobutanoate amidinotransferas...	1,8061E-04
R02120	reaction	Sucrose:1,6-alpha-D-glucan 6-alpha-D-glucosyl...	2,8883E+09
R02381	reaction	Sinapoylcholine sinapohydrolase	6,2208E-04
R02411	reaction	1-alpha-D-Galactosyl-myo-inositol:sucrose 6-a...	1,0000E-06
R02542	reaction	Sphingomyelin ceramide-phosphohydrolase	9,3486E+09
R02573	reaction	Trimethylsulfonium:tetrahydrofolate N-methylt...	4,0782E-02
R02574	reaction	S,S-Dimethyl-beta-propiothetin dimethyl-sulfi...	1,0000E-06
R02922	reaction	Creatinine iminohydrolase	1,6178E-03
R03075	reaction	1-O-(4-Hydroxy-3,5-dimethoxycinnamoyl)-beta-D...	1,3057E-03
R03648	reaction	L-Asparagine:tRNA(Asn) ligase (AMP-forming)	1,0000E-06
R04194	reaction	Sucrose:2,1-beta-D-fructan 1-beta-D-fructosyl...	3,7803E-03
R04212	reaction	Asp-tRNA(Asn):L-glutamine amido-ligase (ADP-f...	1,5690E-02
R04463	reaction	5'-Phosphoribosylformylglycinamide:L-glutamin...	2,8636E-02
R05140	reaction	sucrose:2,6-beta-D-fructan 6-beta-D-fructosyl...	1,3069E+09
R05225	reaction	Adenosyl cobyrinate a,c diamide + 4 L-Glutami...	1,3251E-02
R05362	reaction	2-hydroxy-6-oxoocta-2,4,7-trienoate formylhyd...	2,7852E-03
R05465	reaction	D-Glyceraldehyde 3-phosphate + L-Arginine <=>...	2,8000E-02
R05794	reaction	CDP-diacylglycerol:choline O-phosphatidyltran...	1,0000E-06
R06871	reaction	Phosphocholine phosphohydrolase	1,0000E-06
R07168	reaction	5-methyltetrahydrofolate:NAD+ oxidoreductase	1,3616E-03
R07363	reaction	1,2-dihydroxy-5-(methylthio)pent-1-en-3-one:o...	4,0434E-03
R07377	reaction	Phosphatidylcholine + L-Serine <=> Phosphatid...	1,0000E-06
R07386	reaction	O-1-Alk-1-enyl-2-acyl-sn-glycero-3-phosphoeth...	2,5147E+09
R07409	reaction	choline,reduced-ferredoxin:oxygen oxidoreduct...	1,0000E-06
R07420	reaction	Phosphocreatine <=> Creatinine + Orthophospha...	1,0000E-06
R08197	reaction	L-arginine:pyruvate aminotransferase	1,0000E-06
R08557	reaction	Choline + NAD+ <=> Betaine aldehyde + NADH + ...	1,0000E-06
R08558	reaction	Choline + NADP+ <=> Betaine aldehyde + NADPH ...	1,0000E-06
R08891	reaction	L-glutamine:3-amino-2,3-dideoxy-scyllo-inosos...	4,0254E-02
R08956	reaction	(2S)-2-amino-4-deoxychorismate:L-glutamate am...	5,4339E-03
R09501	reaction	dimethyl sulfide:menaquinone oxidoreductase	2,7131E-02
R09786	reaction	dimethyl sulfide,NADH:oxygen oxidoreductase	1,1807E-04

R10089	reaction	D-ribose 5-phosphate,D-glyceraldehyde 3-phosp...	1,0000E-06
R10243	reaction	Tetrahydrofolate + Acetyl-CoA <=> 5-Methyltet...	3,3562E-04
R10333	reaction	S,S-dimethyl-beta-propiothetin:tetrahydrofola...	1,0000E-06
R10820	reaction	3-(methylthio)propanoate:CoA ligase (AMP-form...	1,3175E-02
R11031	reaction	L-arginine:NAD+ oxidoreductase (deaminating)	1,0000E-06
R11032	reaction	L-arginine:NADP+ oxidoreductase (deaminating)	1,0000E-06
R11033	reaction	L-Arginine <=> N(omega)-Hydroxyarginine	1,0000E-06
R11515	reaction	malonyl-[acyl-carrier protein]:L-glutamine am...	3,0405E-02
R11546	reaction	S-adenosyl-L-methionine:methanethiol S-methyl...	1,1220E-04
R11604	reaction	L-arginine:oxygen oxidoreductase (deaminating...	1,0000E-06
R11711	reaction	L-arginine,reduced-flavodoxin:oxygen oxidored...	6,9781E-04
R11712	reaction	2 L-Arginine + 2 Reduced flavodoxin + 2 Oxyge...	3,5719E-04
R12293	reaction	O-mannose core M3 + ATP <=> Phospho-core M3 +	2,4978E-02
R12352	reaction	N3-Fumaroyl-L-2,3-diaminopropanoate + L-Gluta...	3,1485E-02
R12507	reaction	L-glutamine:4-(methylsulfanyl)-2-oxobutanoate...	2,1967E-04
R12706	reaction	Nopaline + H2O + Acceptor <=> L-Arginine + 2-...	8,3497E-03
R12707	reaction	Octopine + H2O + Acceptor <=> L-Arginine + Py...	9,2854E-03
R12939	reaction	chorismate:L-glutamine aminotransferase (2-am...	3,1199E-03
C00002	compound	ATP	1,9493E-02
C00008	compound	ADP	1,7463E-02
C00009	compound	Orthophosphate	1,1725E-02
C00025	compound	**L-Glutamate**	1,0000E-06
C00031	compound	D-Glucose	4,7394E-02
C00049	compound	L-Aspartate	9,6460E-03
C00062	compound	**L-Arginine**	1,0000E-06
C00064	compound	**L-Glutamine**	1,0000E-06
C00089	compound	**Sucrose**	1,0000E-06
C00095	compound	D-Fructose	1,5823E-03
C00101	compound	Tetrahydrofolate	4,9609E-04
C00114	compound	**Choline**	1,0000E-06
C00152	compound	**L-Asparagine**	1,0000E-06
C00300	compound	Creatine	3,0168E+09
C00440	compound	5-Methyltetrahydrofolate	6,2070E+09
C00511	compound	Acrylic acid	1,8899E+08
C00580	compound	Dimethyl sulfide	1,0000E-06
C00791	compound	Creatinine	1,8990E+07
C02305	compound	**Phosphocreatine**	1,0000E-06
C04022	compound	**S,S-Dimethyl-beta-propiothetin**	1,0000E-06
C08276	compound	3-(Methylthio)propanoate	1,0431E-04

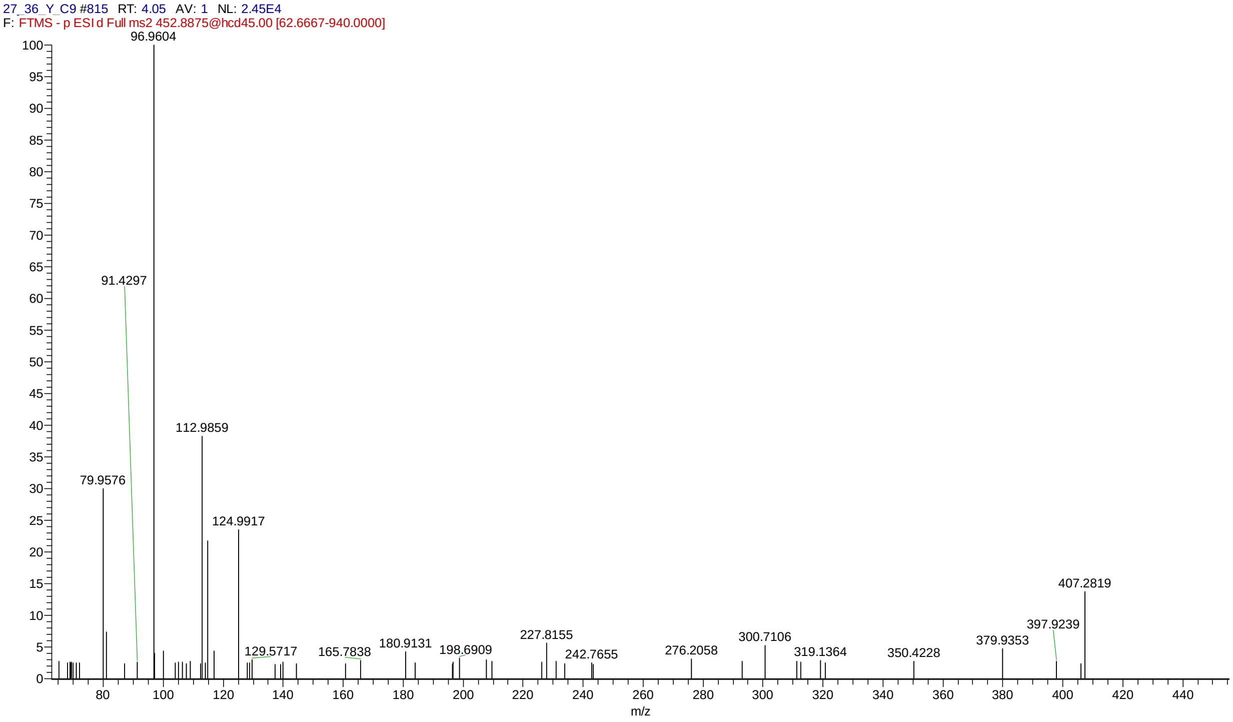

Figure S1. MS^2 spectra of *m/z* 452.8875, $[M+Cl]^-$ adduct of compound 145.

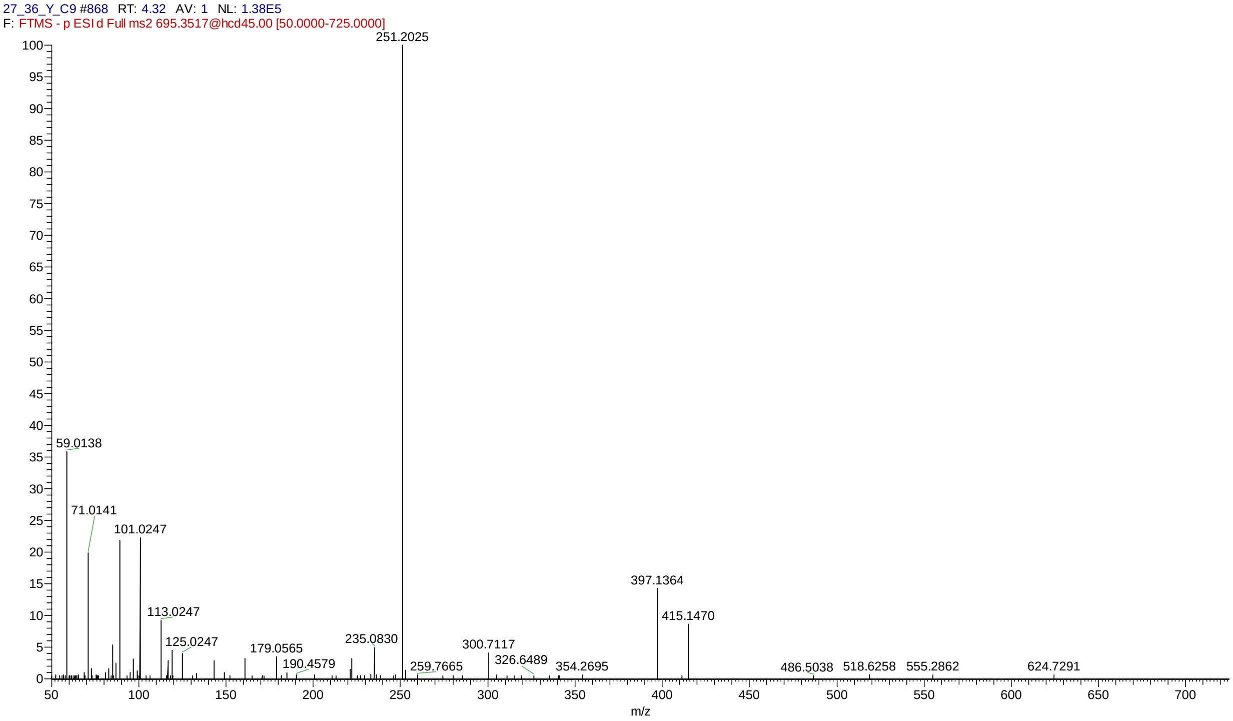

Figure S2. MS^2 spectra of *m/z* 695.3517, $[M-2H+Na]^-$ adduct of compound 9.

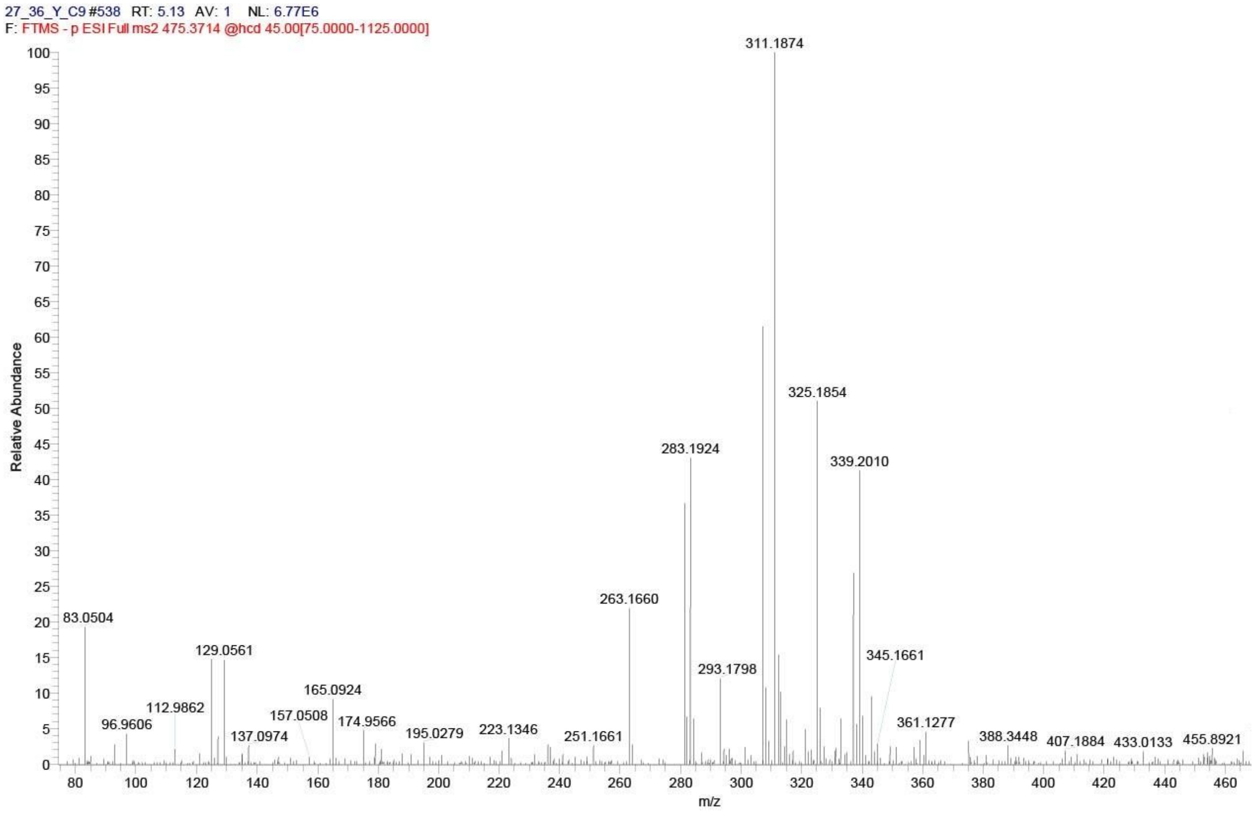

Figure S3. MS^2 spectra of *m/z* 475.3714, $[M+Cl]^-$ adduct of compound 66.

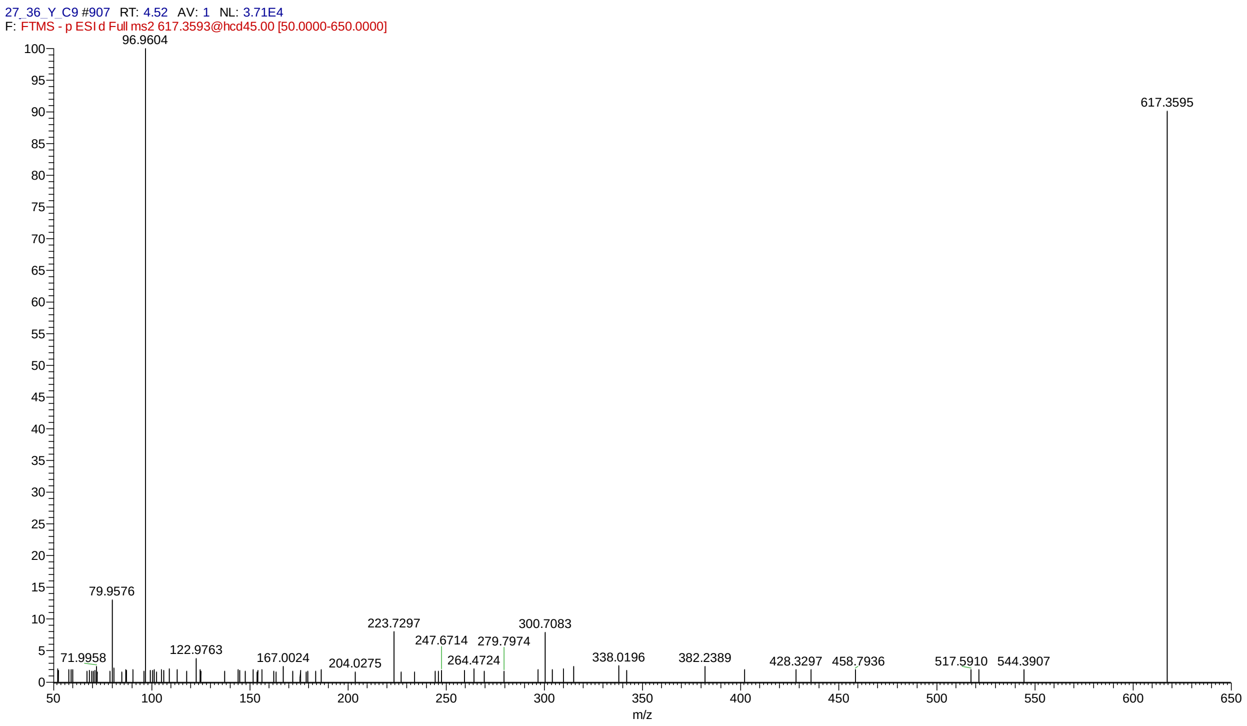

Figure S4. MS^2 spectra of *m/z* 617.3595 (unknown compound 103).

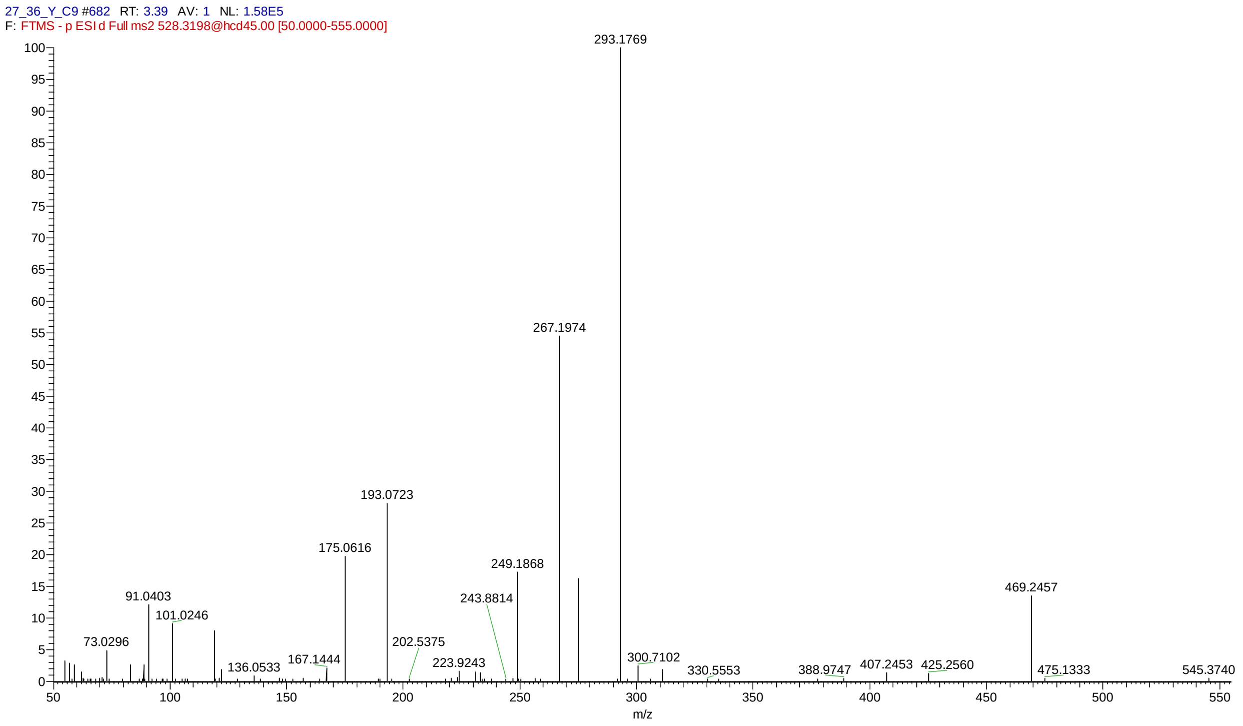

Figure S5. MS^2 spectra of *m/z* 528.3198, $[M-H]^-$ ion of compound 151

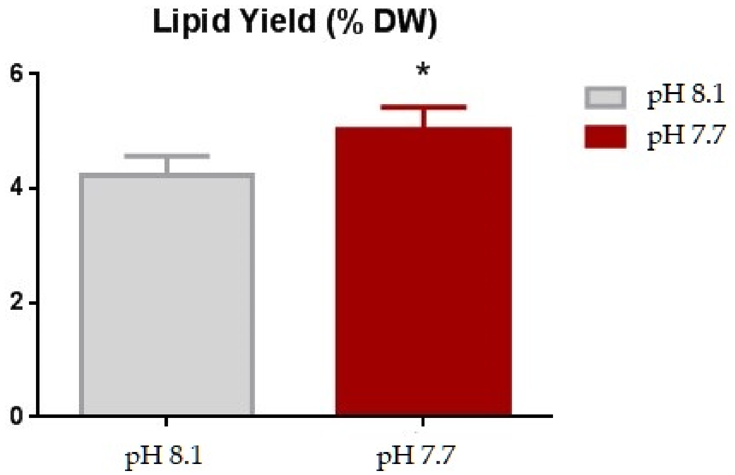

Figure S6. Lipid yield after *Ulva prolifera* extraction by MTBE/MeOH. Values are reported as a percentage (%) of dry weight ± SD of four replicates in the two growth conditions.

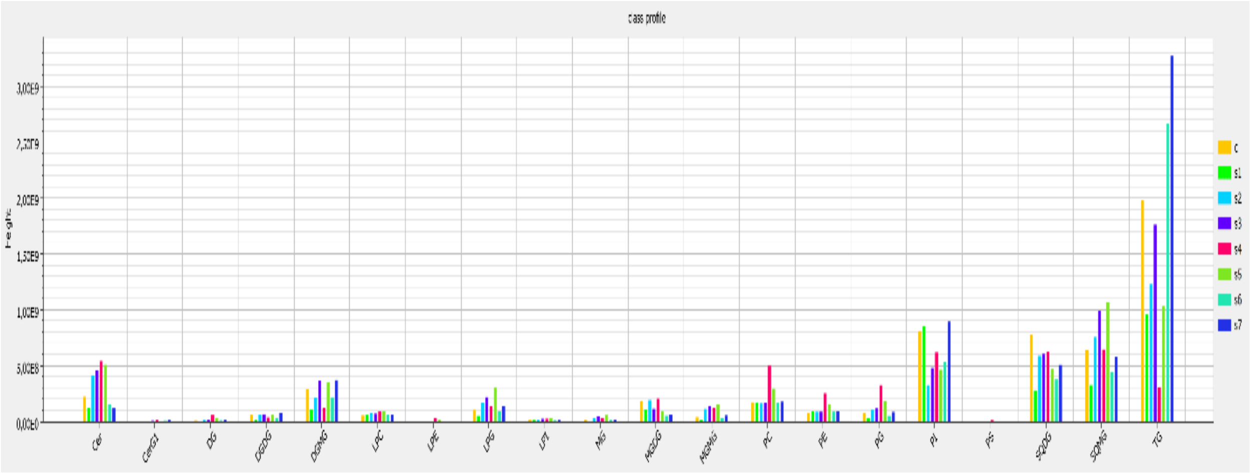

Figure S7. Class profile lipidome of *Ulva prolifera* grown in normal (pH 8.1, replicates c, s1-s3) *vs* acidic (pH 7.7, replicates s4-s7) conditions. The data shown were not normalized.

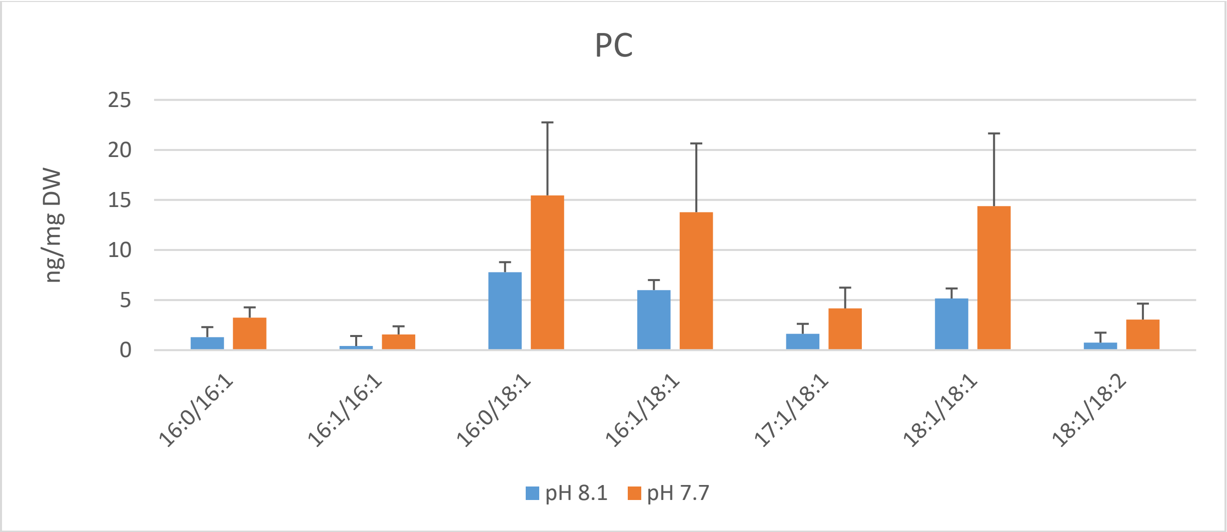

Figure S8. Phosphatidylcholine (PC) composition and semi-quantitation in *Ulva prolifera* grown at pH 8.1 and pH 7.7. Values are reported as ng/mg DW ± SD (n= 4).

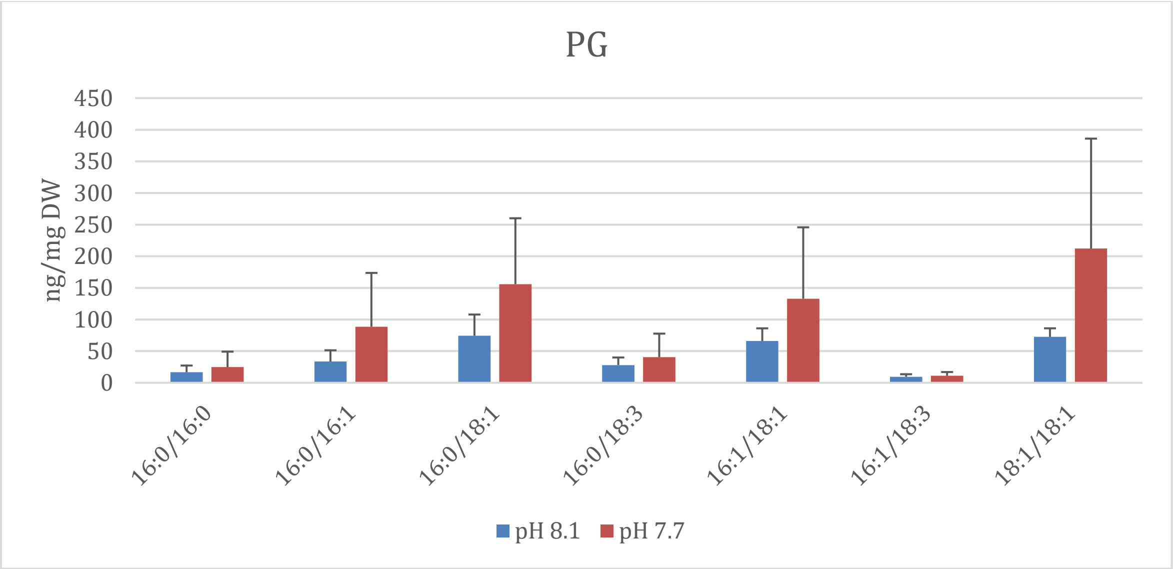

Figure S9. Phosphatidylglycerol (PG) composition and semi-quantitation in *Ulva prolifera* grown at pH 8.1 and pH 7.7. Values are reported as ng/mg DW ± SD (n= 4).

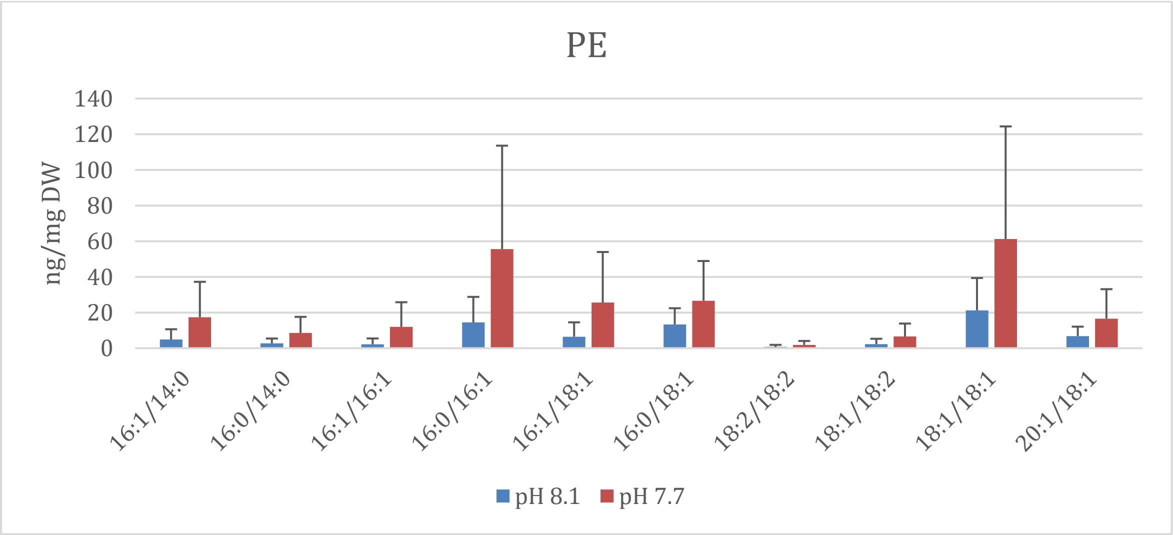

Figure S10. Phosphatidylethanolamine (PE) composition and semi-quantitation in *Ulva prolifera* grown at pH 8.1 and pH 7.7. Values are reported as ng/mg DW ± SD (n= 4).

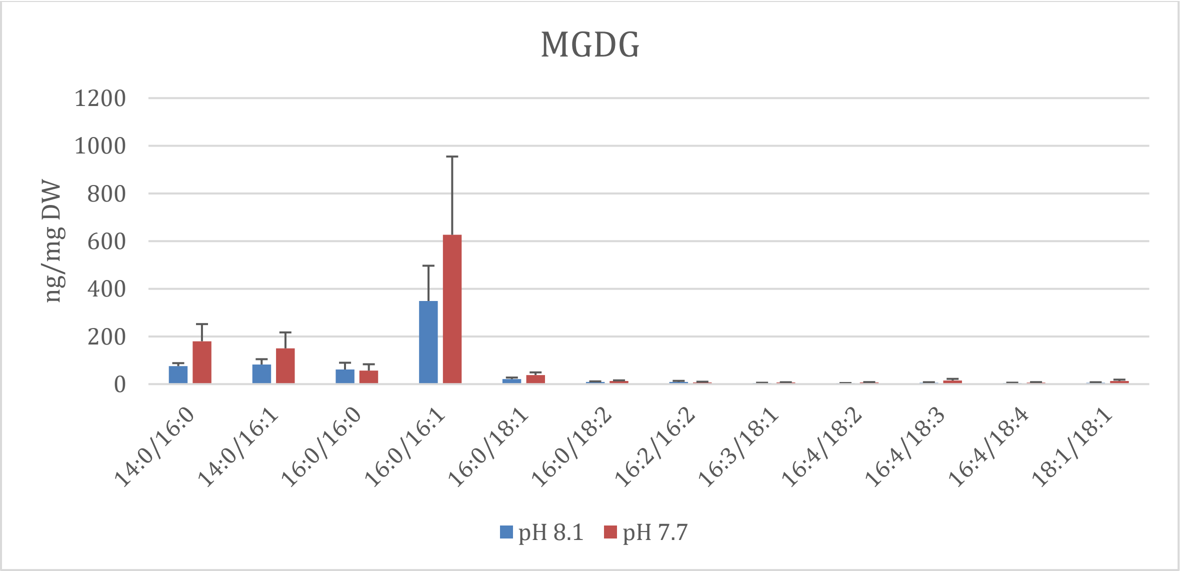

Figure S11. Monogalactosyldiacylglycerol (MGDG) composition and semi-quantitation in *Ulva prolifera* grown at pH 8.1 and pH 7.7. Values are reported as ng/mg DW ± SD (n= 4).

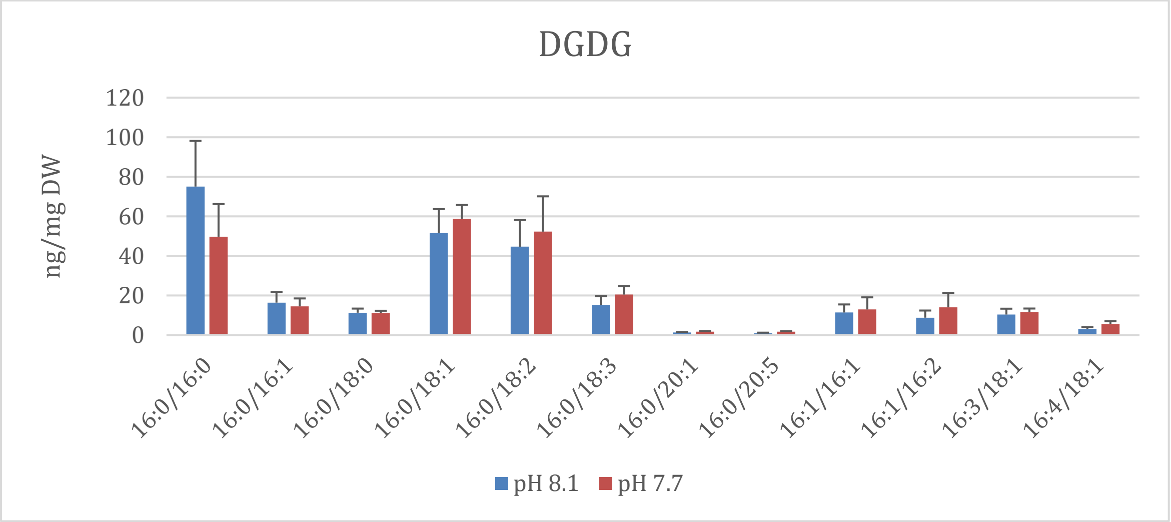

Figure S12. Digalactosyldiacylglycerol (DGDG) composition and semi-quantitation in *Ulva prolifera* grown at pH 8.1 and pH 7.7. Values are reported as ng/mg DW ± SD (n= 4).

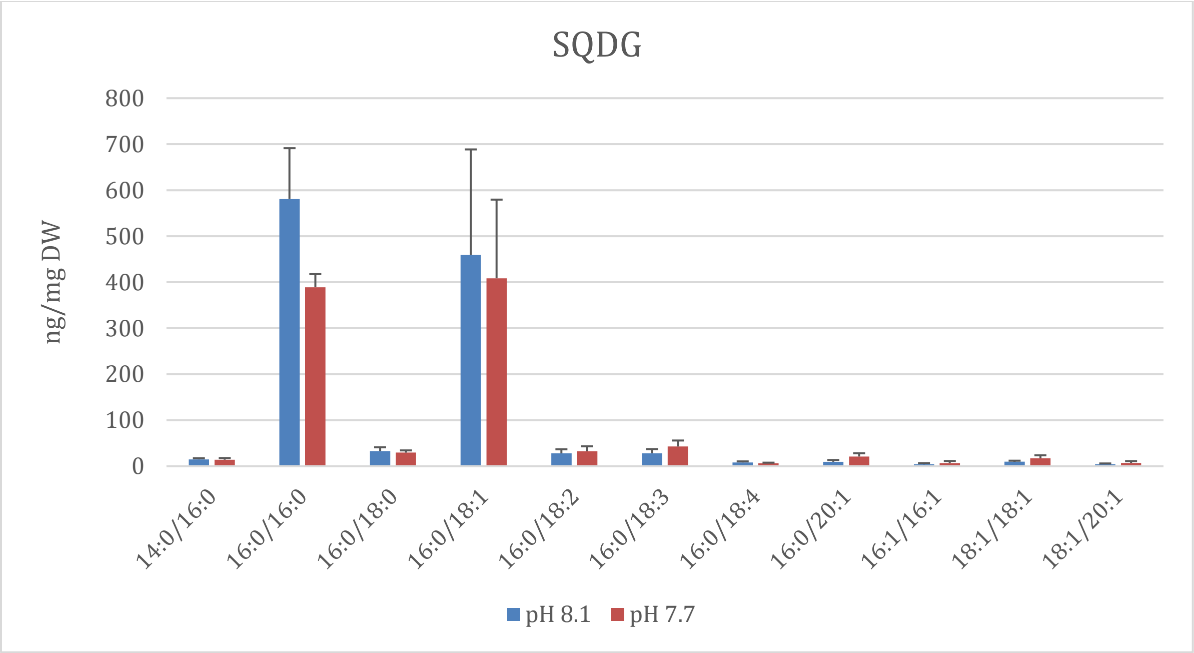

Figure S13. Sulfoquinovosyldiacylglycerol (SQDG) composition and semi-quantitation in *Ulva prolifera* grown at pH 8.1 and pH 7.7. Values are reported as ng/mg DW ± SD (n= 4).

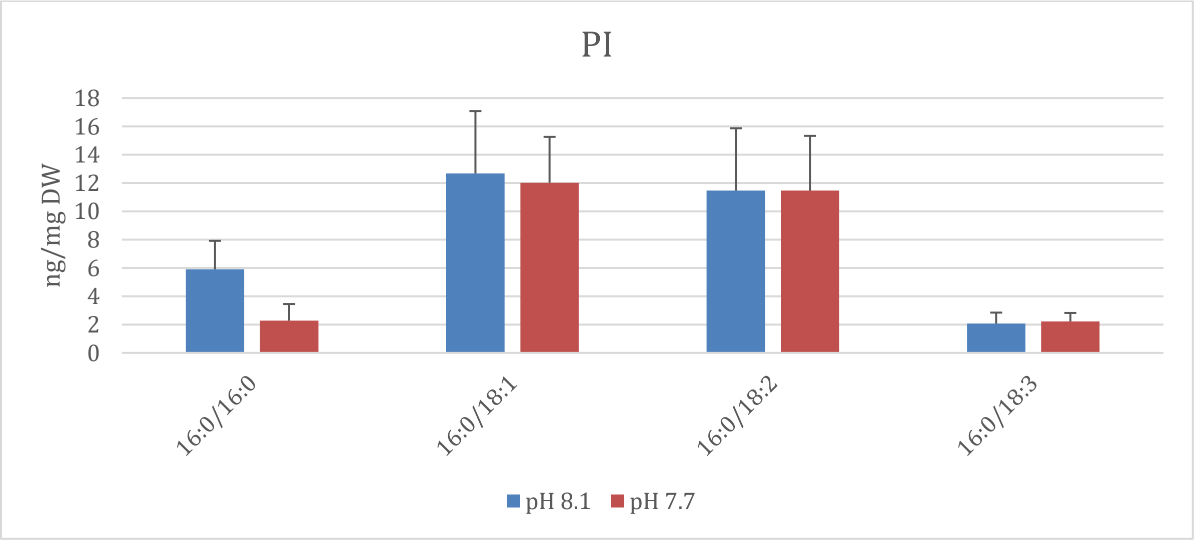

Figure S14. Phosphatidylinositol (PI) composition and semi-quantitation in *Ulva prolifera* grown at pH 8.1 and pH 7.7. Values are reported as ng/mg DW ± SD (n= 4).

Supplementary materials and methods.

Methods S1. Growth curves' logistic equation

Logistic growth curves describe phenomena that start nearly exponential and slow down while reaching the maximum population. The logistic model is defined by a linear decrease of the relative growth rate over time, according to the equation:

$$Y=Y_M*Y_0/((Y_M-Y_0) * \exp(-k*x) +Y_0)$$

Where:

Y_0 is the starting population (same units as Y)

Y_M is the maximum population (same units as Y)

k is the rate constant (inverse units of X)

1/k is the X coordinate of the first inflexion point

At any given time, the growth rate is proportional to Y(1-Y/YM), where Y is the current population size, and YM is the maximum possible population size. As Y approaches the maximum, that second term gets smaller, and the growth slows. The curves obtained for both treatments were compared using a Mann-Whitney test. The differences among slopes of growth curves were evaluated employing confidence intervals using Graphpad Prism (GraphPad Prism version 8.0.0 for Mac, GraphPad Software, San Diego, California USA, www.graphpad.com).

Methods S2. NMR spectra acquisition and parameters

One–dimensional (1D) proton spectra were acquired using the excitation sculpting sequence (Hwang and Shaka, 1995). A double–pulsed field gradient echo was used, with a soft square pulse of 4 ms at the water resonance frequency, with the gradient pulse of 1 ms each in duration, adding 516 transients of 16384 points with a spectral width of 8417.5Hz. Time-domain data were all zero-filled to 32768 points, and prior to Fourier transformation, an exponential multiplication of 0.8 Hz was applied. For the two–dimensional (2D) clean total correlation spectroscopy (TOCSY) (Griesinger et al., 1988; Bax and Davis, 1985) spectra, we used a standard pulse sequence with a spinlock period of 64 ms, achieved with the MLEV–17 pulse sequence, and incorporating the excitation sculpting sequence for water suppression. In general, 256 equally spaced evolution-time period t_1 values were acquired, averaging 64 transients of 2048 points, with 8403.36Hz of spectral width. Time-domain data matrices were all zero-filled to 4096 points in both dimensions, thus yielding a digital resolution of 2.04 Hz/pt. Before Fourier transformation, a Lorentz–to–Gauss window with different parameters was applied for all the experiments' t1 and t2 dimensions. Spectra in water were referred to as internal 0.1 mM TSP, assumed to resonate at δ = 0.00 ppm. Natural abundance 2D ^{1}H–^{13}C heteronuclear single quantum coherence (HSQC) spectra were recorded at 150.90 MHz for ^{13}C, using an echo–anti echo phase-sensitive pulse sequence with adiabatic pulses for decoupling (Palmer III et al., 1991; Kay et al., 1992) and presaturation for water suppression (Schleucher et al., 1994). 256 equally spaced evolution-time period t_1 values were acquired, averaging 96 transients of 2048 points and using GARP4 for decoupling. The final data matrix was zero-filled to 4096 in both dimensions and apodized before Fourier transformation by a shifted cosine window function in t_2 and t_1. The linear prediction was also applied to extend the data to twice its length in t_1. HSQC spectra in water were referred to the α–glucose doublet resonating at 5.24 ppm for ^{1}H and 93.10 ppm for ^{13}C.
